# Structure-function relationships of the disease-linked A218T oxytocin receptor variant

**DOI:** 10.1038/s41380-021-01241-8

**Published:** 2022-01-04

**Authors:** Magdalena Meyer, Benjamin Jurek, Mercedes Alfonso-Prieto, Rui Ribeiro, Vladimir M. Milenkovic, Julia Winter, Petra Hoffmann, Christian H. Wetzel, Alejandro Giorgetti, Paolo Carloni, Inga D. Neumann

**Affiliations:** 1 Department of Behavioral and Molecular Neurobiology, University of Regensburg, Regensburg, Germany; 2 Department of Molecular and Cellular Anatomy, University of Regensburg, Regensburg, Germany; 3 Institute of Neuroscience and Medicine INM-9, Institute for Advanced Simulations IAS-5, Forschungszentrum Jülich, Jülich, Germany; 4 Cécile and Oskar Vogt Institute for Brain Research, University Hospital Düsseldorf, Medical Faculty, Heinrich Heine University Düsseldorf, Düsseldorf, Germany; 5 Department of Biotechnology, University of Verona, Verona, Italy; 6 Department of Psychiatry and Psychotherapy, University of Regensburg, Regensburg, Germany; 7 Regensburg Center for Interventional Immunology (RCI), Regensburg, Germany; 8 Department of Internal Medicine III, University Hospital Regensburg, Regensburg, Germany; 9 Department of Physics, RWTH Aachen University, Aachen, Germany; 10 JARA-Institute: Molecular Neuroscience and Neuroimaging, Institute for Neuroscience and Medicine INM-11/JARA-BRAIN Institute JBI-2, Forschungszentrum Jülich GmbH, Jülich, Germany

**Keywords:** Neuroscience, Psychology, Molecular biology, Autism spectrum disorders, ADHD

## Abstract

Various single nucleotide polymorphisms (SNPs) in the oxytocin receptor (OXTR) gene have been associated with behavioral traits, autism spectrum disorder (ASD) and other diseases. The non-synonymous SNP rs4686302 results in the OXTR variant A218T and has been linked to core characteristics of ASD, trait empathy and preterm birth. However, the molecular and intracellular mechanisms underlying those associations are still elusive. Here, we uncovered the molecular and intracellular consequences of this mutation that may affect the psychological or behavioral outcome of oxytocin (OXT)-treatment regimens in clinical studies, and provide a mechanistic explanation for an altered receptor function. We created two monoclonal HEK293 cell lines, stably expressing either the wild-type or A218T OXTR. We detected an increased OXTR protein stability, accompanied by a shift in Ca^2+^ dynamics and reduced MAPK pathway activation in the A218T cells. Combined whole-genome and RNA sequencing analyses in OXT-treated cells revealed 7823 differentially regulated genes in A218T compared to wild-type cells, including 429 genes being associated with ASD. Furthermore, computational modeling provided a molecular basis for the observed change in OXTR stability suggesting that the OXTR mutation affects downstream events by altering receptor activation and signaling, in agreement with our in vitro results. In summary, our study provides the cellular mechanism that links the OXTR rs4686302 SNP with genetic dysregulations associated with aspects of ASD.

## Introduction

The neuropeptide oxytocin (OXT) regulates multiple social and emotional behaviors, such as social bonding, reciprocal trust, aggression, fear, and anxiety, both in animals and humans [1–3]. Extensive research in rodents revealed endogenous OXT release from hypothalamic neurons within distinct brain regions in response to reproductive [4, 5], stressful [6, 7] or social [8, 9] stimuli, and its binding to intracerebral OXT receptors (OXTR). The OXTR is a G protein-coupled receptor, which is abundantly expressed in the brain [1]. By coupling to different G-proteins [10], the OXTR is linked to multiple intraneuronal signaling cascades, including Ca^2+^, protein kinase C and mitogen-activated protein kinase (MAPK) kinase (MEK1/2) signaling [11, 12], the myocyte enhancer factor 2A (MEF2A) [13], as well as mitochondrial respiration [14]. Some of these pathways, e.g., MEF2A signaling and mitochondrial functioning, have been associated with autism spectrum disorder (ASD) [15–17].

Consequently, brain OXTRs are not only biological targets of endogenous OXT, but potentially also for the treatment of psychopathologies associated with emotional and social deficits such as ASD [18–20]. For example, synthetic OXT, which can be applied intranasally and penetrates brain tissue [21, 22] has been shown to improve social deficits in autistic children [23, 24].

However, the therapeutic efficacy is highly variable across individuals. The cause for this variability is still elusive, but epigenetic modification of OXTR expression [25] or the existence of single nucleotide polymorphisms (SNPs) in the *OXTR* are likely to contribute [26]. Thus, uneven distribution of SNP alleles among clinical trial cohorts could result in the observed variability; consequently, the effect of the mutation on the gene and the gene product is essential knowledge to adequately design clinical studies. So far, the structural as well as functional consequences of SNPs in the gene encoding the OXTR are not fully understood, despite the fact that SNPs in the *OXTR* have already been associated with a plethora of psychological traits in genome-wide association studies (GWAS) [27, 28]. Although most of the described disease-associated SNPs are intronic and/or synonymous mutations [1], non-synonymous SNPs (nsSNPs), which are likely to affect OXTR protein structure and function, have also been associated with severe psychopathological conditions of ASD. For example, the rs4686302 nsSNP has been associated with deficits in social communication and cognition, as well as restricted and repetitive behaviors [29, 30], along with differences in emotional empathy in a non-clinical Chinese cohort [31]. Another study investigated the association of the rs4686302 nsSNP with premature birth as well as reduced Cesarean section prevalence, and found that this variant results in increased contractility upon OXT stimulation in human myometrium biopsies [32].

The nsSNP rs4686302 is located within the coding region of exon 3 in the human *OXTR* gene, leading to an amino acid exchange of alanine to threonine at position 218 (A218T) of the OXTR protein. Intriguingly, in silico sequence-based predictions of the functional significance of this variant did not identify it as damaging [33], in contrast to the observed phenotype. Thus, shedding light on the functional consequences of nsSNP rs4686302 is crucial to assemble a comprehensive model of molecular and intracellular effects that may ultimately affect complex behavioral traits associated with ASD. Recently, the crystal structure of the human OXTR has been solved [34], opening up the avenue to assess such molecular effects in detail.

Here, we transduced HEK293 cells with an N-terminal FLAG-tagged *OXTR* comprising either the reference wild-type (WT) sequence or the rs4686302 nsSNP. Since the OXTR belongs to the family of rhodopsin-like GPCRs that do not rely on a signal peptide [35], but a signal anchor sequence represented by the first transmembrane domain, the N-terminal tag does not interfere with protein translation in the endoplasmic reticulum. By means of FLAG tag-directed fluorescence-activated cell sorting (FACS), we isolated single clones of OXTR-positive cells to create both WT and SNP-containing monoclonal cell lines. To control for genomic effects and to map the insertion site of the *OXTR* gene, we conducted whole-genome sequencing. OXTR protein stability and turnover have also been assessed by cycloheximide degradation assay and compared between nsSNP and WT cells. Because OXTR activation leads to Ca^2+^ release from intracellular stores [36, 37] as well as Ca^2+^ influx from the extracellular space [12], we measured stimulated intracellular Ca^2+^ levels in both cell lines using Ca^2+^ fluorescence imaging. Furthermore, we assessed the activation of the MAPK pathway downstream of the OXTR by western blot, with ERK1/2 being the proposed essential core factor for the anxiolytic and anti-stress effects of OXT [38–40]. As both OXTR-activated pathways signal to the nucleus to regulate gene transcription [41], we also performed RNA sequencing in WT and SNP-containing cells and identified multiple genes, which were found to be differentially regulated by the presence of the SNP. The impact of the mutation on the receptor structure and on downstream intracellular events was finally investigated by molecular and systems biology modeling. The computational results have been validated by comparison with the aforementioned protein degradation and Ca^2+^ imaging assays. Altogether, the combination of in vitro and in silico approaches used in this work allows to analyze the molecular and cellular effects of the OXTR A218T variant and opens the way for future rational drug design efforts.

## Material and methods

### Cell culture

HEK293 cells (provided by Prof. Eugen Kerkhoff, University Hospital Regensburg) were cultured in DMEM (#D8437, Sigma Aldrich, Darmstadt, Germany), supplemented with 10% heat-inactivated Gold fetal bovine serum (#FBS-HI-11A, Capricorn, Germany), and penicillin/streptomycin (#P4333, Sigma Aldrich) at 37 °C/5% CO_2_ until 80% confluency. Passaging was performed at least once a week by gentle trypsinization. Cells were tested for mycoplasma contamination on a regular basis.

### Transduction of HEK293 cells with *OXTR* gene variants

For a stable integration of the *OXTR* variants in the genome, moloney murine leukemia virus (MMLV) vectors were designed (VectorBuilder, Neu-Isenburg, Germany), and HEK293 cells were transduced according to the manufacturer’s protocol. The constructs contained the respective human *OXTR* gene (transcript variant 1, accession number NM_000916.3) or the SNP-containing variant (rs4686302) and an N-terminal 3xFLAG tag conjugated to the gene.

### Establishment of monoclonal cell lines expressing the OXTR

To reduce variability, we used monoclonal cell lines. For single cell sorting, cells were stained with an anti-DDDDK tag antibody that recognizes the 3xFLAG tag, conjugated to phycoerythrin (ab72469, abcam, Cambridge, UK) and sorted on a BD FACSAria^TM^ IIu high-speed cell sorter (Becton Dickinson, Heidelberg, Germany).

### Whole-genome sequencing

Two cell lines, expressing either the reference or the SNP-containing *OXTR* gene, were chosen based on their expression levels of the OXTR protein validated by 3xFLAG tag western blot. Further analysis, including the genomic integration site of the constructs, whole-genome sequencing, and subsequent bioinformatics analyses were performed by CeGaT GmbH (Tübingen, Germany). For details, please see Tabs. S1–S4.

### Protein isolation and western blot

Protein extraction and western blotting was performed as previously described in Meyer et al. [13]. Due to a lack of specific OXTR antibodies [42], we detected OXTR expression via the 3xFLAG tag. Specific antibodies for anti-DDDDK (ab49763, abcam, Cambridge, UK), ERK1/2 (9102, Cell Signaling Technology, Frankfurt am Main, Germany) and pERK1/2 (4370, Cell Signaling Technology) revealed expression levels of the OXTR, and total and phosphorylated ERK1/2, respectively. The total protein loading was controlled by the “Stain-Free” method from Bio-Rad (Feldkirchen, Germany). Uncropped images of western blots are provided in Fig. S1. All protein quantifications by western blot were replicated at least 3 times.

### Flow cytometry

For analysis of cell surface expression of the OXTR, cells were stained with anti-DDDDK tag antibody conjugated to phycoerythrin (ab72469, abcam, Cambridge, UK) and DAPI (Sigma-Aldrich, Munich, Germany) to exclude dead cells. Data were acquired on a BD FACSCelesta™ (Becton Dickinson, Heidelberg, Germany) and analyzed with FlowJo^®^ v9.9.6 (Treestar Inc., Ashland, OR, USA).

### Cycloheximide protein degradation assay

The turnover of the OXTR protein was determined using cycloheximide (CHX)-mediated inhibition of de novo protein synthesis. Cells were seeded and treated with 20 μg/ml CHX (Sigma, Taufkirchen, Germany) for 0–24 h. Proteins were isolated with RIPA, and western blot analysis was performed. The expression of 3xFLAG was analyzed in whole cell lysates.

### Cytosolic Ca^2+^ imaging with Fura-2-AM

The basal cytosolic Ca^2+^ amount and the OXT-induced cellular Ca^2+^ response were assessed using the ratiometric Ca^2+^ indicator Fura-2-AM. Solutions (Ringer ± Ca^2+^) and OXT (final concentration 100 nM) were applied with a perfusion system. Regions of interest were drawn over selected cells in the visual field using the Zen imaging software (ZEISS) and FIJI/ImageJ. Ca^2+^ traces were plotted with the Origin Software (OriginLab, version 9.7.0.188) evaluating the basal cytosolic Ca^2+^, area under the curve, amplitude, as well as full width at half maximum.

### Molecular modeling

Both monomeric and homodimeric forms of OXTR may be present at the OXTR expression levels in the HEK293 cellular lines studied here [43]. Therefore, both forms were modeled.

Our structural models of WT and A218T OXTR monomeric proteins were based on the X-ray structure of the OXTR A218T variant monomer (PDB code 6TPK) [34], which corresponds to an inactive state. We used SwissModel [44] to revert eight thermo-stabilizing mutations present in the crystallographic construct [34] and replace the fusion protein by the sequence of the human OXTR intracellular loops (UniProtID P30559). The residue at position 218 was modeled as either alanine or threonine using the Rotamers tool [45] in the UCSF Chimera program [46]. Models of the WT and A218T monomeric proteins in an intermediate and in the active states were either generated with SwissModel [44] or obtained from GPCRdb [47] (Tab. S5). The change in folding free energy (ΔΔG_fold_) of the OXTR monomer upon the mutation was evaluated by analyzing our A218T models with the mCSM-membrane [48], DynaMut [49], DynaMut2 [50], and PremPS [51] servers. The corresponding change in vibrational entropy (ΔΔS_vib_) was estimated using DynaMut [49]. ΔΔG_fold_ and ΔΔS_vib_ are correlated, in a qualitative way, with changes in monomer stability and flexibility upon mutation, respectively [52–54]. For details, please see Tab. S7.

The OXTR/OXTR homodimer models were built using a structural superposition strategy similar to that in reference [55]. Several dimer interfaces are possible in class A GPCRs [56, 57], such as the OXTR [55, 58]. Here, we focused on the TM5/TM5’ dimer, for which the A218T variant is expected to have a more significant effect. We compiled all the experimental structures of GPCR dimers with a TM5 interface (Tab. S6) available in the DIMERBOW database [56]. Then, we superimposed our OXTR A218T monomer model onto each of the monomers of the experimental dimeric structures by using the MatchMaker tool [59] in the UCSF Chimera program [46]. The thus-generated A218T OXTR/OXTR dimer models were used to build the corresponding WT OXTR-OXTR dimer models by reverting in silico the Thr218 mutation back to Ala using the Rotamers tool [45]. The different homodimer models were ranked based on the OXTR/OXTR binding free energy (ΔG_bind_) evaluated using the PRODIGY webserver [60, 61]. We then estimated the change in OXTR/OXTR binding energy upon mutation (ΔΔG_bind_), using the SAAMBE-3D [62], MutaBind2 [63], and mCSM-PPI2 [64] servers. All free energy and entropy calculations reported here are highly approximate and used for qualitative comparisons only (Tabs. S7–S10).

### Systems biology modeling

Our mathematical model of the OXT-mediated Ca^2+^ release from the endoplasmic reticulum integrates a previous model for the serotonin 2A receptor [65] with a kinetic model for intracellular Ca^2+^ oscillations [66] (see Supplementary Material for details). Simulation parameters are described in Tab. S11, the reaction equations and parameters for each of the signaling cascade steps are described in Tab. S12 and S13.

Our model was developed under the PySB framework [67] and integrated using the SciPy ODE numerical integrator [68]. The results were analyzed using NumPy [69], SciPy [68] and scikit-learn [70] libraries. All simulations and analysis codes were written and run in a Jupyter notebook [71] (see https://github.com/rribeiro-sci/OXTR.git).

### RNA sequencing and gene ontology analysis

The transcriptome of three samples of each OXTR cell line (WT and A218T mutant) in response to stimulation with 100 nM OXT for 1 h was analyzed by RNA Sequencing and Gene Ontology (GO) [72, 73] annotation of differentially regulated genes (SRA SAMN21439292, SAMN21439293, CeGaT GmbH, Tübingen, Germany).

The list of genes associated with ASD was downloaded from the SFARI database [74] (https://gene.sfari.org/autdb/GS_Home.do) and compared to the list of differentially regulated genes obtained from the transcriptome analysis. Additionally, genes were categorized according to the gene scoring module of SFARI. Genes scored as category 1 show high confidence, i.e. they have been clearly implicated in ASD and meet the most rigorous threshold of genome-wide significance. Category 2 represents strong candidates that are uniquely implicated by GWAS reaching genome-wide significance and most likely with a functional effect. Category 3 contains genes with suggestive evidence of ASD correlation from significant, yet unreplicated, studies.

### Statistical analysis

Data were analyzed either by *t*-test or two-way ANOVA followed by Tukey post hoc test (Sigma Plot, version 13.0, Systat Software). The variance between groups was similar, and statistical significance was accepted at *p* < 0.05. Due to the large number of genes in the RNA sequencing data set, Benjamini–Hochberg multiple-testing correction was used for controlling false discovery rate (*p*_adj_, adjusted *p* value). In Ca^2+^ imaging experiments, n represents number of cells/traces, in western blots, n represents number of cell lysates. Data are presented as mean ± standard error of the mean.

## Results

**Fig. 1:**
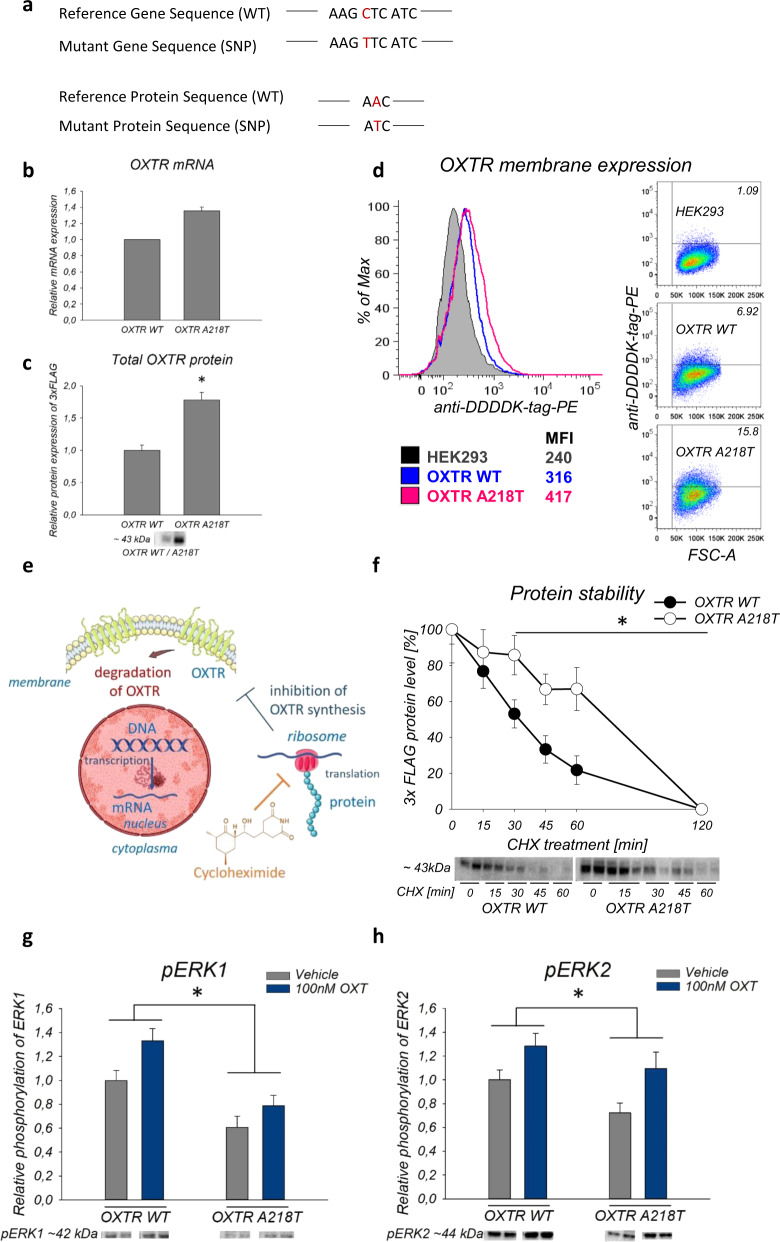
Inherent properties and intracellular effects of the wild-type (WT) and A218T oxytocin receptor (OXTR). **a** Gene and protein sequence of WT and A218T OXTR. **b** Relative mRNA expression levels of the OXTR in A218T compared to WT cells analyzed by RNA sequencing. *p_adj_ < 0.001. **c** *T*-test revealed a significantly increased protein expression of the 3xFLAG tag in the whole cell lysate of A218T compared to WT cells. *n* = 6. *p = 0.005. **d** Flow cytometric determination of the surface expression level of the OXTR in WT and A218T cells. Values inside the flow cytometry plots represent the percentage of cells above and beyond a set threshold. Colored values under the histogram plot show the mean fluorescence intensity (MFI). FSC-A: forward scatter area. **e** Scheme of cycloheximide (CHX) inhibition effect on protein translation. The schematic art pieces used in this figure were provided by Servier Medical Art (https://smart.servier.com) licensed under a Creative Commons Attribution 3.0 Unported License. **f** Quantification of 3xFLAG levels after CHX treatment as percentage of the initial protein level (0 min of CHX treatment). Two-way ANOVA revealed a significant effect between the cell lines in the protein stability evaluated by CHX assay. *n* = 4 and 6 for each data point. *p (cell line) = 0.002. *p (treatment) < 0.001. **g**, **h** Quantification of western blot results showing relative phosphorylation level of ERK1 and ERK2 in the OXTR WT and A218T cell line after stimulation with 100 nM oxytocin (OXT) for 1 h. Direct comparison of the cell lines revealed that the OXTR A218T cells show a lower phosphorylation level than the OXTR WT cells independent of the treatment (Vehicle = gray bars/OXT = blue bars). *p < 0.049. *n* = 10 per group. **b**, **c**, **f**, **g**, **h** Data are shown as mean ± SEM. Band intensity was normalized by whole lane staining using the “Stain-Free” method (© Bio-Rad).

The molecular and cellular consequences of cellular expression of the *OXTR* nsSNP rs4686302 were assessed using a combination of molecular biology techniques complemented with computational modeling. First, HEK293 cells (which do not express the OXTR endogenously) were transduced with a construct containing either the WT or OXTR A218T variant, combined with an N-terminal 3xFLAG tag (Fig. 1a). Next, positively transduced cells were sorted to establish two monoclonal cell lines. Whole-genome sequencing revealed that the WT *OXTR* construct was inserted on chromosome 8 within the *MYC* gene (see Tab. S1), whereas the A218T mutant *OXTR* construct was inserted twice on chromosome 6 at two positions (see Tab. S1), which are located within the *NUDT3* and *USP45* gene, respectively. For each insertion site, we extracted ten genes upstream and downstream of the location and analyzed potential insertion-induced dysregulations. These genes (see Tab. S2) were not considered for further analysis.

The double gene insertion in the OXTR A218T cell line resulted in a 1.36-fold upregulation of the *OXTR* transcript, which was confirmed by RNA sequencing, and a 1.78-fold upregulation of the protein in whole cell lysates compared to the OXTR WT-expressing cells (Fig. 1b, c and Tab. S3, S4). Whole cell lysates include vesicular OXTR [75] and potentially even nuclear OXTR [76]. However, the functionally most important fraction is the cell surface-bound OXTR, whose level was determined by flow cytometry. Both cell lines showed only a low expression level of the OXTR in the membrane, which was slightly higher in the A218T compared to the WT cells (Fig. 1d).

The stability of the OXTR variants was analyzed by the CHX chase assay (Fig. 1e). 3xFLAG levels were quantified after treatment with CHX as percentage of the initial FLAG protein level (0 min of CHX treatment). We revealed a significantly reduced protein degradation turnover, i.e. higher stability, of the OXTR A218T variant compared with WT (Fig. 1f).

Subsequent analyses of MAPK pathway activation, i.e. of basal and OXT-induced phosphorylation of ERK1/2, were performed by western blot in both OXTR WT and mutant cells (Fig. 1g, h) [40]. We found a significantly reduced MAPK activation in the A218T cells as reflected by both pERK1 (Fig. 1g) and pERK2 (Fig. 1h) levels after 60 min OXT stimulation.

**Fig. 2:**
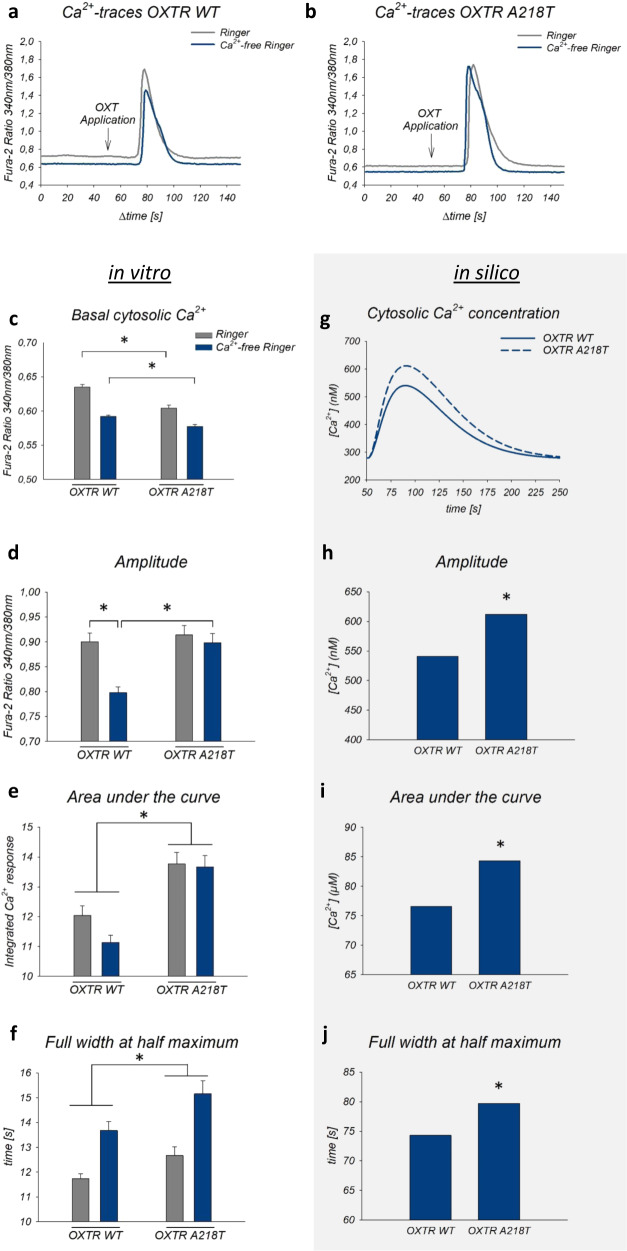
Intracellular Ca^2+^ dynamics in the oxytocin receptor (OXTR) wild-type (WT) and A218T variant in vitro and in silico. **a**, **b** Representative Ca^2+^-traces of OXTR WT or A218T cells upon stimulation with 100 nM oxytocin (OXT) in Ca^2+^-containing (gray line) or Ca^2+^-free (blue line) Ringer’s solution. Ca^2+^ levels were analyzed as fluorescence ratio at 510 nm after excitation at 340 and 380 nm. **c** Basal cytosolic Ca^2+^ levels of OXTR WT and A218T cells reflected by Fura-2 340 nm/380 nm ratios under both conditions. *p < 0.001. **d** Mean amplitude of OXT-induced Ca^2+^-signals in Ca^2+^-free and Ca^2+^-containing Ringer’s solution in WT and mutant cells. *p < 0.001. Interaction between cell line and treatment (± Ca^2+^) *p = 0.01. **e** Mean area under the curve calculated as integral over time above baseline in OXTR A218T compared to OXTR WT cells under both conditions. *p < 0.001. **f** Two-way ANOVA revealed a main effect between the cell lines regarding the full width at half maximum (FWHM). *p = 0.02. **g** Graphical representation of the simulation curves of Ca^2+^ concentration upon stimulation with OXT in Ca^2+^-free Ringer’s solution. **h** Maximal amplitude of OXT-induced Ca^2+^ simulation curves’ peaks of OXTR WT and OXTR A218T. **∗** ratio = 1.13. **i** Area under simulation’s curves of OXTR WT and A218T. **∗** ratio = 1.10. **j** FWHM of the OXT**-**induced Ca^2+^ simulation’s curves of OXTR WT and OXTR A218T. **∗** ratio = 1.07. **c**–**f** Bars show mean + SEM in presence (gray bars) or absence (blue bars) of extracellular Ca^2+^. Two-way ANOVA with sample size for graphs: *n* (OXTR WT + /−Ca^2+^) = 91/96, *n* (OXTR A218T + /−Ca^2+^) = 63/89.

Additionally, in vitro evaluation of cellular Ca^2+^ responses using Fura-2 Ca^2+^ imaging (Fig. 2a, b) revealed reduced basal cytosolic Ca^2+^ levels in A218T cells compared to WT, both in the presence and absence of extracellular Ca^2+^ (Fig. 2c). However, the amplitude of the OXT-induced Ca^2+^ signal was higher in A218T cells compared with WT cells incubated in Ca^2+^-free Ringer (Fig. 2d). The area under the curve revealed a cell line-specific effect showing a higher increase in the cytosolic Ca^2+^ concentration upon OXT stimulation in A218T compared to WT cells (Fig. 2e). The full width at half maximum, which reflects the kinetics of the OXT-induced Ca^2+^ response, also differed significantly between the two cell lines irrespective of the bathing solution, indicating a prolonged OXT-induced Ca^2+^ response in the A218T cells (Fig. 2f).

**Fig. 3:**
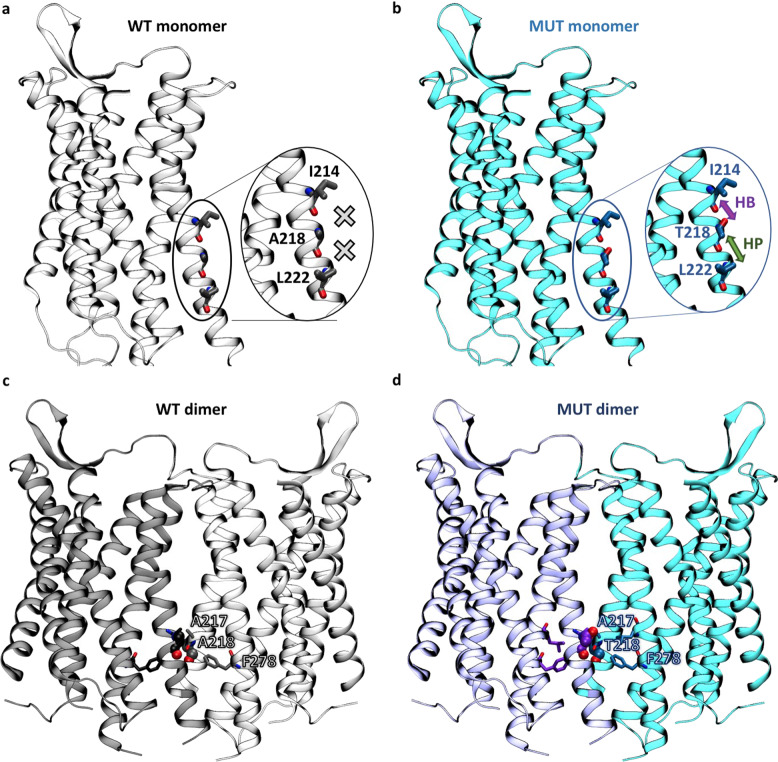
Comparison of the local environment of residue 218 in wild-type (WT) and mutant (MUT) models based on the crystal structure of the OXTR A218T variant (PDB code 6TPK). **a**, **b** Monomeric forms (inactive state). No interactions, hydrogen bonds (HBs) and hydrophobic interactions (HPs) involving the residue are indicated by a cross, a violet arrow, and a green arrow, respectively. The counterpart homology models of the active and intermediate states are shown in the Supplementary Information (Fig. S2). **c**, **d** Top homodimeric models with a TM5 interface based on the experimental structure of the μ-opioid receptor dimer (PDB code 4DKL). The residue in position 218 is shown as spheres and residues surrounding it within 5.5 Å as sticks. The other OXTR/OXTR models are shown in the SI (Fig. S3).

To provide a rationale for the increased stability and the differential OXTR-mediated Ca^2+^ responses in the A218T variant, we resorted to in silico methods. OXTR can exist as monomer and homodimer [43, 55]; therefore, the effect of the mutation was investigated for both forms. The X-ray structure of OXTR A218T [34] was solved in its monomeric form and shows that the side chain of T218, located in the transmembrane helix 5 (TM5), establishes intrahelical interactions with Ile214 and Leu222 (see Figs. 3 and S2). In contrast, in our model of the monomeric OXTR WT, the A218 side chain does not form any interactions (Figs. 3 and S2). The additional intramolecular interactions present in the A218T variant may stabilize the monomeric receptor, as also suggested by a change in folding free energy predicted by several web servers (see Tab. S7).

To assess the effect of the A218T variant on the OXTR/OXTR homodimer, we focused on the TM5/TM5’ interface, where residue 218 is located. In our top-ranked models of the WT OXTR homodimer (Figs. 3 and S3), A218 does not form any intermolecular interactions across the protein/protein interface. Instead, in the mutant homodimer, T218 forms hydrophobic interactions with residues of the adjacent monomer in two out of the top three dimer models (Figs. 3 and S3). The additional intermolecular interactions in the T218A variant may be associated with an increase in dimer stability, as indicated by the change in OXTR-OXTR binding free energy predicted with several web servers (see Tabs. S8–S10). Therefore, our modeling predicts that the mutant A218T OXTR is more stable, in line with the experimental in vitro data presented in Fig. 1d. Nonetheless, our calculations only provide a (qualitative) estimation of thermodynamics stabilities, which differ from the experimental readout related to protein degradation.

The change in intrahelical interactions at the OXTR monomer level is also predicted to result in a decrease in protein flexibility in the A218T variant compared to WT (Tab. S7). This is in line with previous computational studies showing that the presence of Ser or Thr in TM helices affects their dynamics [77]. Nonetheless, it should be noted that the calculations presented here account only in a very simplified way for the contribution of protein dynamics (see Supplementary Material). Molecular dynamics simulations, in combination with energetic analyses, would be needed to estimate more accurately the mutational effects on protein stability and flexibility [78–80].

Taken together, the effects of the A218T variant on both stability and flexibility of monomeric and dimeric OXTRs (Tabs. S7 and S10), albeit small, may affect the participation of the TM5 helix in receptor activation [81–84]. In turn, this may impact the subsequent OXTR-mediated signaling pathways. Here, we explored this possibility by building a mathematical model of the OXTR-dependent effects on the intracellular Ca^2+^ concentration (in Ca^2+^-free Ringer’s solution). Importantly, such model integrates the previous molecular modeling data in the parameter that implicitly depends on receptor activation: the kinetic constant (kf_coupling_wt and kf_coupling_mut for the WT and A218T variant, respectively, Tab. S11) describing the binding between the receptor and its cognate G-protein (see reaction 3 in Tab. S12). By changing this single parameter on passing from WT to A218T (see kf_coupling_wt and kf_coupling_mut in Tab. S11), the in silico model (Fig. 2g) turns out to reproduce the experimental Ca^2+^ concentration curves (Fig. 2c), including the maximal amplitude (Fig. 2h), the area under the curve and the full width at half maximum (Fig. 2i, j). Thus, within its limitations (see Supplementary Material), our approach further supports the change in receptor activation caused by the mutation as a key factor for the observed changes in intracellular Ca^2+^ concentrations.

**Fig. 4:**
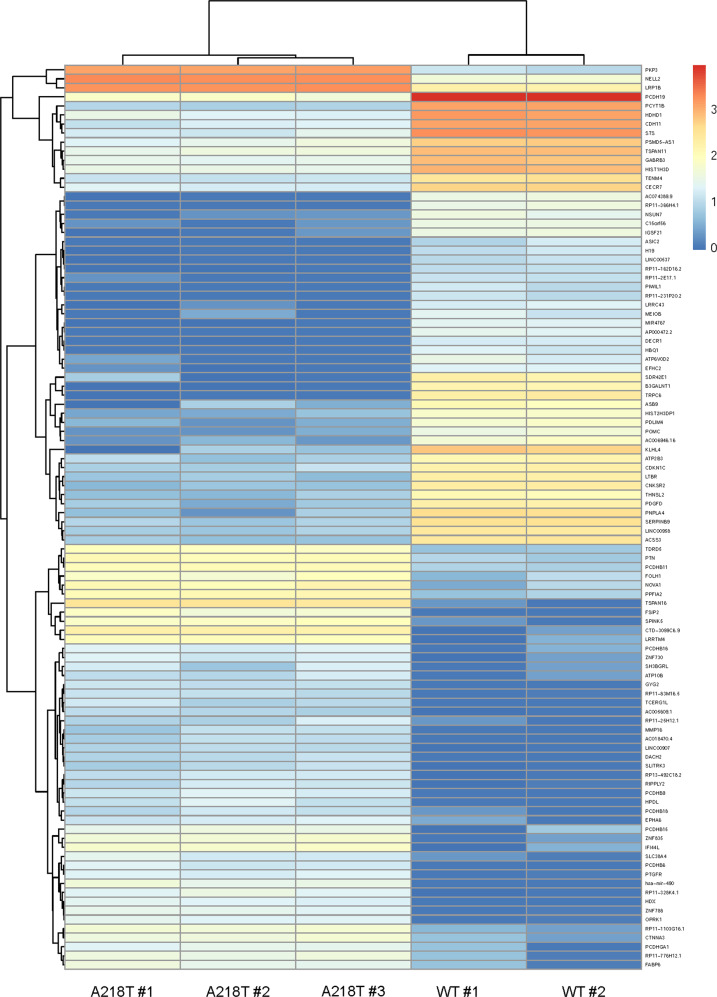
Heatmap of the top 100 differentially expressed genes in the oxytocin receptor (OXTR) A218T variant compared to OXTR wild-type (WT) cells. Color-coded values represent the log2FoldChange expression after normalization of the biological replicates in each cell line. Genes (rows) and cell lines (columns) were clustered hierarchically according to similarity between expression levels.

The aforementioned changes in Ca^2+^ dynamics and MAPK pathway in the OXTR mutant cells are likely to affect gene expression. Indeed, RNA sequencing analyses revealed a considerable cohort of differentially regulated genes in OXT-stimulated A218T *versus* WT cells. When filtered by corrected significance *p*_adj_ < 0.05, a total of 7823 genes were differentially regulated with the top 100 differentially expressed genes being listed in a heatmap (Fig. 4). Out of these genes, 429 have been identified as ASD risk genes after comparison with the database provided by SFARI. Additionally, classification of the filtered genes according to the gene scoring module in SFARI showed that 107 differentially expressed genes fall in category 1 of the ASD scoring, of which the 50 most frequently reported are listed in Tab. 1.

**Table. 1:** List of the 50 most frequently reported ASD-associated genes, which are differentially expressed in OXTR A218T compared to WT cells.

Genes are indicated with full gene name, the number (#) of reports, and log2FoldChange from transcriptome analysis of the cell lines. With a color code from red to blue, positive values indicate a higher expression in A218T compared to WT and negative values reduced expression.

In order to identify the underlying biological functions, in which the differentially expressed genes are implicated, we performed a gene set testing. The GO analysis was conducted for the differentially expressed genes between OXTR WT and A218T cell lines with a log2FoldChange cutoff of 1.5. The analysis was done using either only the upregulated genes or only the downregulated genes, or both upregulated and downregulated genes together, as summarized in Tab. S3. We were able to identify Ca^2+^ and MAPK signaling GO terms, which validates the in vitro/in silico results obtained in this study. In addition, other terms that were previously associated with the OXTR [13, 14], such as cellular morphology and connectivity, or mitochondrial functioning, were selected from the dataset and assigned in the Supplementary Material (see Tab. S4).

## Discussion

In this study, we evaluated the functional relevance and cellular consequences of a specific genetic variation in the *OXTR* gene, the nsSNP rs4686302 (Fig. 1a). In humans, this A218T mutation of the OXTR has been associated with cognition deficits, differences in emotional empathy, and preterm birth [29–31, 33]. Our approach, using MMLVs to permanently integrate the *OXTR* gene into the HEK293 genome, provided monoclonal cell lines with stable receptor expression levels. Using whole-genome sequencing, we mapped the integration site(s) of the *OXTR* (Tab. S1) and identified a set of unintendedly disrupted adjacent genes that were excluded from further analyses (Tab. S2). The double insertion of the OXTR A218T construct resulted in a higher level of mRNA (Fig. 1b) and total cellular protein, compared to the WT OXTR cells (Fig. 1c). Additionally, we found that there is a discrete difference in the cell surface expression of the OXTR between the two cell lines (Fig. 1d). Such minor increases in surface expression could be functionally relevant, as gene duplications have already been associated with diagnosed ASD. For instance, a case study associated an *OXTR* gene duplication with pervasive developmental disorder, especially obesity and behavioral issues [85].

The nsSNP rs4686302 is located within the coding sequence of Exon 3 and leads to an alanine/threonine amino acid exchange. We found that the A218T mutation has a stabilizing effect on the receptor protein. Using CHX protein degradation assays, we showed that the OXTR A218T variant exhibits significantly longer half-life kinetics (Fig. 1f) suggesting an increased protein stability. In support, molecular modeling indicated that the A218T mutation stabilizes the OXTR monomeric structure relative to WT (Tab. S7) by forming additional stabilizing intramolecular interactions (Figs. 3 and S2). The A218T mutation may further stabilize the protein in the homodimeric state (Tab. S10) by establishing intermolecular interactions across the OXTR/OXTR interface (Figs. 3 and S3).

Comparison of the downstream signaling cascades between the two cell lines revealed a more pronounced OXT-induced increase in intracellular Ca^2+^ levels in OXTR A218T relative to WT cells. Moreover, the kinetic profile of the Ca^2+^ response indicated a prolonged signal duration in mutant cells.

Our calculations further suggest that the A218T mutation, located in TM5 (Fig. 3), is associated with decreased flexibility of this helix, which is likely to affect receptor activation and, hence, the response of OXTR-triggered intracellular signaling pathways. Indeed, mathematical modeling suggests that altered activation kinetics of the receptor affects intracellular Ca^2+^ concentrations in a manner compatible with the in vitro results. However, it remains to be clarified, whether the elevated OXT-induced Ca^2+^ levels in OXTR A218T cells affect cellular viability or induce, when prolonged, cytotoxicity [86]. Interestingly, WT and A218T cells also differed with respect to the Ca^2+^ source they mainly rely on: whereas influx from the extracellular space dominates in OXTR WT cells, release from intracellular Ca^2+^ stores seems to be the main source in OTXR A218T cells. Since several Ca^2+^-gated membrane channels of the OXTR A218T cell line are downregulated (Fig. 4 and Tab. 1), using intracellular Ca^2+^ sources might have compensatory reasons. Particularly noteworthy in this context is the downregulation of the receptor potential canonical 6 (*TRPC6)* channel, accompanied by a similar, but less pronounced, downregulation of the related *TRPC3*. TRPC3 forms a complex with TRPC6 [87], and disruption of TRPC6 expression and protein function has been associated with ASD [88]. Moreover, activation of the TRPC6 channel promotes dendritic growth via the CAMKIV/CREB pathway [89], a pathway known to be coupled to the OXTR [39] and to OXT-induced neurite growth regulation [13, 14]. Furthermore, a link between TRP channel activation and the OXT system has previously been described [12].

Moreover, the alpha 1C subunit of the voltage-gated L-type calcium channel (*CACNA1C*), which is among the most prominent ASD-associated genes, was found to be downregulated by a factor of 2.17 in OXTR A218T cells (Table 1). Thus, the reduced Ca^2+^ permeability may explain the lower basal levels of cytosolic Ca^2+^ in OXTR A218T cells. The observed differences in Ca^2+^ dynamics might play an important role in maintaining downstream signal specificity, e.g. in the MAPK cascade. Indeed, stimulation with OXT resulted in a less pronounced MAPK pathway activation in OXTR A218T compared with WT cells. Since the anxiolytic effect of the OXT is mediated via phosphorylation of ERK1/2 [38, 40], we hypothesize that the attenuated phosphorylation level in OXTR A218T cells accounts, to some extent, for comorbid anxiety found in some cases of ASD.

Furthermore, the diminished activity of the ERK1/2 pathway impacts transcriptional regulation by downstream transcription factors. We have mapped changes in the transcriptome of OXTR WT *versus* OXTR A218T by means of RNA sequencing. As both cell lines derived from the same mother-cell line, changes in the transcriptome can be traced back to the induced genomic alterations. The transcriptome analysis revealed a large cohort of significantly regulated genes in OXT-stimulated OXTR A218T compared to WT cells. When filtered by corrected significance *p*_adj_ < 0.05, 7823 genes were found to be differentially regulated, of which 429 have been associated with ASD risk, providing a potential molecular link between the A218T variant and a psychopathological phenotype.

## Conclusions

Various SNPs in the OXTR, including the A218T mutation, have been associated with psychological traits and psychopathologies. Our in vitro and in silico results provide a starting point to understand the molecular, intracellular and functional consequences of an expression of the OXTR A218T variant, which has been associated with ASD symptoms. The expression of the variant turns out to result in (i) enhanced receptor stability, possibly by forming additional intra- and intermolecular interactions, (ii) altered intracellular Ca^2+^ dynamics, likely by affecting receptor activation, and (iii) other downstream effects including changes in MAPK activation and expression of several ASD-related target genes. Thus, allosteric ligands that reverse the observed effects of the A281T mutation on the receptor’s activation may provide a potential therapeutic strategy for ASD patients bearing the nsSNP rs4686302.

## Supplementary information

Supplemental Material and Results

Supplementary Figure S1
