# Supplementary material for: Structure-function relationships of the disease-linked A218T oxytocin receptor variant
Source: Mol Psychiatry. 2022 Jan 4;27(2):907–17. doi: 10.1038/s41380-021-01241-8 (PMC9054668; doi:10.1038/s41380-021-01241-8)
Supplement: Supplementary file 1 — Supplemental Material and Results [file 41380_2021_1241_MOESM1_ESM.docx]

## Supplementary information

### **Transduction of HEK293 cells with *OXTR* gene variants**

The *OXTR* gene, which was placed in-between the two long terminal repeats during vector construction, is thereby permanently inserted into the host genome, alongside the rest of the viral genome. Vector construction, amplification and packaging has been executed by VectorBuilder (Neu-Isenburg, Germany). To examine the transduction efficiency and the further inheritance of the transduced genome, we employed a control virus expressing EGFP. Genetically unmodified HEK293 cells served as reference for all transductions.

### **Establishment of monoclonal cell lines expressing the OXTR**

Transduced cells were deposited individually into the wells of a 96-well plate and re-cultured for several weeks using a mixture of fresh and conditioned medium. Conditioned medium was generated by cultivating HEK293 cells in normal growth medium to near confluency. The medium containing growth factors released by the cell layer was removed, sterile-filtered, and stored at 4°C until further use.

**Protein isolation and Western Blot**

For the extraction of proteins, cells were scraped in 1 mL ice-cold PBS using a cell scraper (#83.1830, Sarstedt, Nümbrecht, Germany), centrifuged and resuspended in 100 µl RIPA lysis buffer (R0278, Sigma Aldrich, Darmstadt, Germany) supplemented with HALT inhibitor and EDTA (78444, Thermo Fisher, Waltham, MA) to obtain whole cell lysates.

20 to 30 µg of whole cell extract were loaded and separated in a 12% Mini PROTEAN or Criterion TGX Stainfree gel (Bio-Rad, Feldkirchen, Germany), respectively. Semi-dry blotting of separated proteins to nitrocellulose membranes was conducted using the FAST Blotter by Bio-Rad. Stainfree total protein method by Bio-Rad served as protein loading control.

### **Cytosolic Ca^2+^ imaging with Fura-2-AM**

7 × 10^5^ cells were seeded on sterile glass coverslips (diameter 25 mm), placed in 6-well plates, and grown overnight in medium, at 37°C humidified air and 5% CO_2_. On the next day, cells were loaded with 2 µM Fura-2-AM and Pluronic F127 in OptiMEM (Life Technologies, Carlsbad, USA) for 30 min at 37 °C, humidified air and 5% CO_2_. For imaging, the loading medium was replaced by assay buffer (140 mM NaCl, 5 mM KCl, 1.8 mM CaCl_2_, 1 mM MgSO_4_, 10 mM glucose, and 10 mM HEPES) and the coverslip was mounted in a chamber on the inverted microscope (ZEISS Observer Z.1, Jena, Germany) equipped with a Fluar 40/1.3 objective lens (ZEISS). Cells were illuminated with light of 340 and 380 nm wavelength (BP 340/30 HE, BP 387/15 HE) using a fast wavelength switching and excitation device (Lambda DG-4, Sutter Instrument, Novato, USA), and fluorescence was detected at 510 nm (BP 510/90 HE and FT 409) using an AxioCam MRm LCD camera (ZEISS).

### **RNA Sequencing and gene ontology analysis**

For RNA sequencing, trimmed raw reads were aligned to the human reference genome (hg19) using STAR (version 2.5.2b). Differential expression analysis between groups was performed with DESeq2 (version 1.24.0) in R (version 3.6.1) (R Core Team 2015). DESeq2 uses a negative binomial generalized linear model to test for differential expression based on gene counts.

Using the R-package GOfuncR^1^, we identified significantly enriched and depleted GO terms for up- and down-regulated genes, separately as well as in the combined dataset. GOfuncR uses a hypergeometric test to identify over- and underrepresented GO terms in a set of genes given the distribution of GO terms in the background (e.g. the human transcriptome). For GO analysis (CeGaT GmbH; Tübingen, Germany) significantly down- and up-regulated genes were extracted using a log2 fold cutoff of 1.5.

## Results Whole Genome Sequencing Analysis

**Tab. S1: Identified insertion points of the oxytocin receptor (OXTR) gene constructs in human HEK293 cells.**

| **Cell line** | **Chromosome** | **Position** | **Gene** |
| --- | --- | --- | --- |
| OXTR WT | 8 | 128799928 | MYC |
| OXTR A218T | 6 | 34292480 | NUDT3 |
|  | 6 | 99962623 | USP45 |

**Tab. S2: Differentially expressed genes close to the insertion points and their p-value.**

| **Insertion point** | **Gene** | **Adjusted p-value** |
| --- | --- | --- |
| MYC | MYC | 5.24e-33 |
|  | FAM49B | 0.025 |
|  | LPCAT1 | 7.66e-23 |
|  | FAM84B | 0.0005 |
|  | ASAP1 | 0.01 |
| NUDT3 | SCUBE3 | 1.16e-5 |
|  | SNRPC | 0.006 |
|  | GRM4 | 0.0007 |
|  | ITPR3 | 1.35e-32 |
|  | UQCC2 | 0.004 |
|  | HMGA1 | 6.36e-16 |
| USP45 | MMS22L | 0.04 |
|  | POU3F2 | 0.04 |
|  | FBXL4 | 5.73e-5 |
|  | COQ3 | 1.73e-5 |
|  | PNISR | 5.99e-26 |
|  | USP45 | 0.02 |
|  | CCNC | 1.94e-7 |
|  | SIM1 | 7.04e-5 |
|  | ASCC3 | 0.0001 |


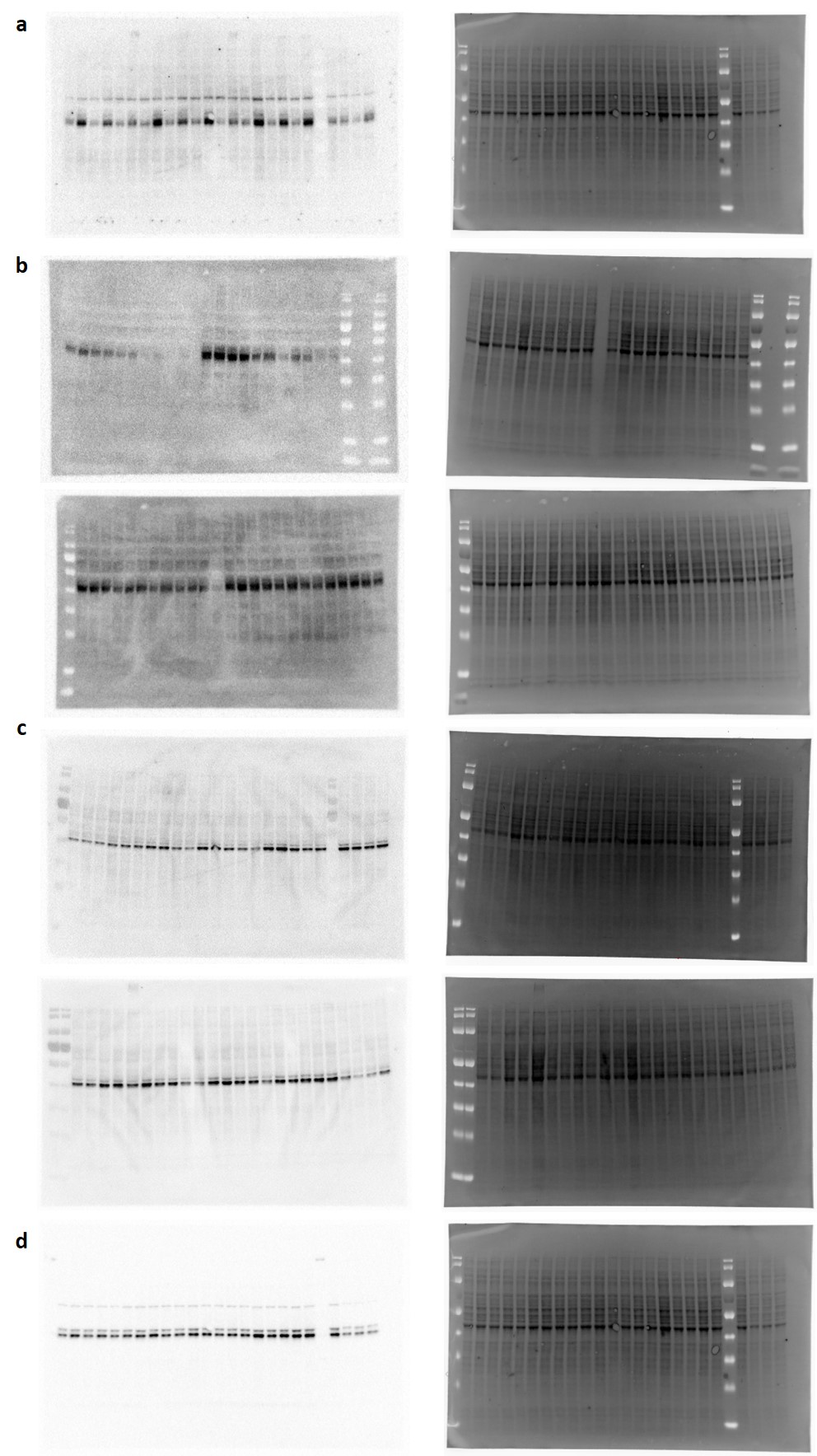


**Fig. S1: Uncropped Western Blot images shown in Fig. 1. Blots on the left side show the respective antibody image and blots on the right side the corresponding stain-free blot image for total protein loading control. a,** Uncropped images of total OXTR expression Blot (Fig. 1c). **b,** Uncropped images for blots of the Cycloheximide Assay showing the 3x-FLAG expression (Fig. 1f). **c,** Uncropped images of pERK1/2 blots (Fig. 1g and 1h). **d,** Uncropped images of ERK1/2 total blot. The groups did not show significant differences in the expression of ERK1/2 total.

## GO analysis of the differentially expressed genes between WT and A218T OXTR expressing cell lines

**Tab. S3:** **Number (#) of analyzed genes and enriched gene ontology (GO) terms.** GO terms were identified for genes significantly up- and down-regulated in OXTR A218T compared to WT cells separately, as well as in the combined dataset.

| **Dataset** | **# of significant genes** | **Genes with GO annotation (in %)** | **Biological processes** | **Cellular components** | **Molecular functions** |
| --- | --- | --- | --- | --- | --- |
| Up-regulated | 804 | 61.94 | 50 | 14 | 19 |
| Down-regulated | 1803 | 81.2 | 306 | 92 | 26 |
| Both | 2607 | 75.26 | 286 | 90 | 23 |

Based on the results obtained in this study and in previous publications of our group^2-4^, a few GO terms were selected from the dataset and assigned to OXTR-coupled processes (Tab. S4).

**Tab. S4: Selection of gene ontology (GO) terms up- and downregulated in oxytocin receptor A218T *versus* wildtype cells with identification number.**

|  | GO term | GO ID |
| --- | --- | --- |
| Receptor binding and stability | oxytocin receptor binding | GO:0031855 |
|  | cellular response to cycloheximide | GO:0071409 |
| Calcium signaling | calcium-mediated signaling | GO:0019722 |
|  | cellular response to calcium ion | GO:0071277 |
|  | calcium ion homeostasis | GO:0055074 |
|  | calcium ion transport | GO:0006816 |
|  | calcium ion transmembrane import into cytosol | GO:0097553 |
|  | calcium ion transmembrane transport | GO:0070588 |
|  | calcium ion transmembrane transport via high voltage-gated calcium channel | GO:0061577 |
|  | calcium ion transmembrane transport via low voltage-gated calcium channel | GO:0090676 |
| MAPK signaling | MAPK cascade | GO:0000165 |
|  | p38 MAPK cascade | GO:0038066 |
|  | activation of MAPK activity | GO:0000187 |
|  | activation of MAPKK activity | GO:0000186 |
|  | activation of MAPKKK activity | GO:0000185 |
| Connectivity | cell-cell adhesion | GO:0098609 |
|  | cell-cell adhesion mediated by integrin | GO:0033631 |
|  | cell-cell signaling | GO:0007267 |
|  | integrin-mediated signaling pathway | GO:0007229 |
| Cellular morphology | cytoskeleton organization | GO:0007010 |
|  | actin cytoskeleton organization | GO:0030036 |
|  | intermediate filament organization | GO:0045109 |
|  | microtubule cytoskeleton organization | GO:0000226 |
|  | cell morphogenesis | GO:0000902 |
|  | dendrite extension | GO:0097484 |
|  | axon extension | GO:0048675 |
| Mitochondrial function | mitochondrial calcium ion homeostasis | GO:0051560 |
|  | mitochondrial calcium ion transmembrane transport | GO:0006851 |
|  | mitochondrial ATP synthesis coupled electron transport | GO:0042775 |
|  | mitochondrial ATP synthesis coupled proton transport | GO:0042776 |
|  | mitochondrial respiratory chain complex assembly | GO:0033108 |

## Molecular modeling

At the relatively high OXTR expression levels in the HEK293 cellular lines studied here, both monomeric and homodimeric forms of OXTR may be present^5, 6^. Therefore, we modeled both forms. Hereafter, we describe the computational methodologies used for predicting the structural effects of the A218T variant on the two OXTR forms. The results obtained are discussed in part here and in part in the main text.

## Methods

*OXTR monomer structure.* The structural model of OXTR A218T in the inactive state was based on the X-ray structure of the OXTR A218T variant (PDB code 6TPK)^7^, in complex with the antagonist retosiban (Chart S1). Retosiban, as well as the allosteric modulators cholesterol and magnesium present in the crystal structure, were removed from our structural models. None of these ligands are located near residue 218. In addition, the crystal structure contains eight thermostabilizing mutations and a fusion protein. Therefore, we used SwissModel^8^ to revert such mutations and replace the fusion protein by the sequence of the human OXTR intracellular loops (UniProtID P30559).

The structural model of OXTR WT in the inactive state was converted into the OXTR A218T model by mutating *in silico* Ala218 to Thr using the Rotamers tool^9^ in the UCSF Chimera program^10^. As a result, the structures of the OXTR A218T and WT receptor models are identical, except for position 218.


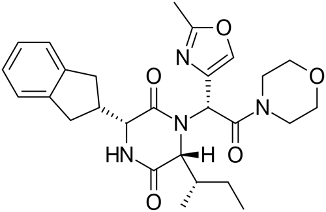


**Chart S1. Chemical structure of retosiban (Vaccinationist, Public domain, via Wikimedia Commons).**

We then generated OXTR models in different GPCR activation states (active state, one of the possible intermediate states and inactive state) by homology modeling (HM). Two different HM approaches were used. On the one hand, we used SwissModel^8^ to generate models based on a single template. On the other hand, we retrieved multitemplate-based models already precomputed in the GPCRdb^11^; the ones described here correspond to version 2020-07-30. All the template structures used correspond to class A GPCRs (i.e. the same class as OXTR) and are listed in Tab. S5. They were selected based on their sequence identity with OXTR and their activation state, as classified in the GPCRdb^11^. Namely, the active, intermediate and inactive states are defined on the basis of the 7TM Open IC distance, which indicates the openness of the intracellular side, where the G-protein coupling occurs upon receptor activation. This parameter is larger than 7.15 Å for active, between 2 and 7.15 Å for intermediate and below 2 Å for inactive state structures^11^. In order to facilitate the comparison among structures, this distance is normalized with respect to fully active state structures, which are defined to be 100% degree active. Hence, inactive and intermediate states show low and in-between percentage values, respectively.

**Tab. S5: Templates used to build the OXTR monomeric models.**

| **Structural template** | **PDB code** | **Resolution (Å)** | **Sequence identity (%)^*^** | **Degree Active (%)** | **State** | **Models^#^** |
| --- | --- | --- | --- | --- | --- | --- |
| OXTR A218T | 6TPK | 3.2 | 94.2 | 14 | Inactive | A-B |
| **SwissModel HM templates** | **PDB code** | **Resolution (Å)** | **Sequence identity (%)*** | **Degree Active (%)** | **State** | **Models^#^** |
| Orexin 1 receptor | 6TP6 | 2.3 | 24.2 | 1 | Inactive | C-D |
| Cysteinyl leukotriene receptor 1 | 6RZ5 | 2.5 | 18.9 | 50 | Intermediate | G-H |
| Muscarinic acetylcholine receptor 2 in complex with G_o_-protein | 6OIK | 3.6 | 20.8 | 100 | Active | K-L |
| **GPCRdb HM main templates** | **PDB code** | **Resolution (Å)** | **Sequence identity (%)*** | **Degree Active (%)** | **State** | **Models^#^** |
| OXTR A218T | 6TPK | 3.2 | 94.2 | 14 | Inactive | E-F |
| Serotonin 5-HT_2B_ receptor | 4IB4 | 2.7 | 18.1 | 76 | Intermediate | I-J |
| Rhodopsin | 4ZWJ | 3.3 | 18.1 | 95 | Active | M-N |

^*^ Sequence identity based on HHblits alignment^12^. Note that the sequence identity of the human OXTR A218T X-ray structure^7^ and the target sequence of human OXTR WT is slightly below 100% due to the presence of eight thermostabilizing mutations in the experimental structure, besides the A218T mutation.

^#^ The labels used for the models are the same as in Fig. S2. The first label indicates the WT model and the second the A218T mutant counterpart.

*OXTR Dimer.* Class A GPCRs, including OXTR^6, 13^, have been proposed to form dimers using different TM interfaces^14, 15^. The residue in position 218 is located at TM5 and, thus, the A218T variant may either have a direct effect on the TM5/TM5’ dimer (in which it is part of the protein/protein interface) or affect indirectly the dimers exhibiting other TM interfaces. Here we focused on the OXTR/OXTR homodimers involving TM5, the helix where the residue in position 218 is located. These TM5/TM5’ homodimers are expected to be affected more significantly by the A218T variant. The experimental structures of GPCR dimers with a TM5 interface were retrieved from the DIMERBOW database^14^ (version March 5th, 2020) and are listed in Tab. S6. We analyzed those dimeric structures using the PRODIGY^16, 17^ and PRODIGY-CRYSTAL webservers^18, 19^. All fourteen TM5/TM5’ dimer templates turned out to have a favorable protein/protein binding free energy (Tab. S6). Almost all of them (11 out of 14) were predicted to be realistic biological complexes (Tab. S6). Nonetheless, the probability of the prediction for the three dimers classified as crystallographic is below 0.7. Hence, we decided not to discard any template and use them all for our structural alignment.

**Tab. S6: Class A GPCR dimer X-ray structures involving TM5 at the protein/protein interface present in the DIMERBOW^14^ database (version: March 5^th^, 2020).**

| **GPCR** | **PDB code** | **Number of interactions^*^** | **Interface**  **Prediction^#^** | **ΔG_bind_**  **(kcal/mol)^§^** |
| --- | --- | --- | --- | --- |
| Squid Rhodopsin (OPS-01) | 2Z73 | 28 | XTAL (0.668) | -6.8 |
| Squid Rhodopsin (OPS-02) | 2Z73 | 30 | BIO (0.524) | -3.4 |
| Human CXC Chemokine receptor type 4 (CXCR4) | 3ODU | 42 | BIO (0.772) | -5.2 |
| Turkey β1-adrenergic receptor (ADRB1-01) | 4BVN | 32 | BIO (0.704) | -4.1 |
| Mouse μ-opioid receptor (OPRM) | 4DKL | 76 | BIO (0.908) | -8.5 |
| Human P2Y purinergic receptor (P2Y12) | 4NTJ | 13 | BIO (0.516) | -3.8 |
| Turkey β1-adrenergic receptor (ADRB1-02) | 5A8E | 32 | BIO (0.716) | -4.4 |
| Human β2-adrenergic receptor (ADRB2) | 5JQH | 17 | XTAL (0.504) | -3.9 |
| Human A_2A_ Adenosine receptor (AA2AR-01) | 5NM4 | 20 | BIO (0.596) | -5.2 |
| Human A_2A_ Adenosine receptor (AA2AR-02) | 5OLO | 36 | XTAL (0.636) | -6.0 |
| Human A1 adenosine receptor (AA1R) | 5UEN | 44 | BIO (0.752) | -5.1 |
| Human Platelet-activating factor receptor (PTAFR) | 5ZKQ | 21 | BIO (0.616) | -3.5 |
| Human Prostanoid receptor (PE2R3) | 6AK3 | 28 | BIO (0.580) | -4.2 |
| Human 5-hydroxytryptamine 2C (5HT2C) | 6BQG | 62 | BIO (0.852) | -5.6 |

^*^ Number of pair-wise residue contacts, i.e. atom pairs of the two monomers closer than 5.0 Å, obtained from the DIMERBOW database^14^ and the PRODIGY-CRYSTAL webtool^18, 19^.

^#^ The dimer interface is predicted to be biological (BIO) or crystallographic (XTAL) using the PRODIGY-CRYSTAL webtool^18, 19^. The probability of the classification is indicated between parentheses, with values closer to one indicating the likelihood of the dimer to belong to the BIO or XTAL class, i.e. biological or crystallographic complex, respectively.

^§^ Calculated with the PRODIGY webserver^16, 17^.

Following the procedure in reference^6^, we superimposed our OXTR A218T monomer model based on the X-ray structure^7^ onto each of the monomers of the experimental dimeric structures in Tab. S6. The structural alignment was performed using the MatchMaker tool^20^ in the UCSF Chimera program^10^. The thus-generated A218T OXTR/OXTR dimer models were used to build the corresponding WT OXTR/OXTR dimer models by reverting *in silico* the Thr218 mutation back to WT Ala using the Rotamers tool^9^. Altogether, we generated 28 models, fourteen for the A218T homodimer and fourteen for the WT homodimer. All our OXTR/OXTR dimer models are in the inactive state. We did not model the active and intermediate states because the position of the TM5 helix remains approximately the same along GPCR activation^21, 22^, in spite of the repacking of the TM5 and TM6 helices.

The OXTR dimeric models were analyzed using the same webserver tools as the experimental GPCR dimer templates. PRODIGY-CRYSTAL^18, 19^ was used to predict whether the OXTR/OXTR TM5/TM5’ interface is expected to be biological or crystallographic. The protein-protein binding free energy (ΔG_bind_) of the OXTR homodimeric models was evaluated using the PRODIGY webserver^16, 17^.

*Effect of the A218T variant on the OXTR models.* We used several webserver-based tools to estimate, at a qualitative level^23, 24^, the effect of the A218T variant on the OXTR monomeric and homodimeric structural models.

The effect on OXTR monomer stability was estimated qualitatively by calculating the change in protein folding free energy upon mutation (ΔΔG_fold_). To have a consensus prediction, we used several webservers, namely mCSM-membrane^25^, DynaMut^26^, DynaMut2^27^ and PremPS^28^. In addition, we investigated a possible effect on monomer flexibility by calculating the change in vibrational entropy (ΔΔS_vib_) using DynaMut^26^. The OXTR A218T monomeric models were used as input, and the webservers reverted the Thr218 mutation back to the WT Ala.

The effect on OXTR/OXTR dimer stability was estimated qualitatively by calculating the change in protein-protein binding free energy upon mutation (ΔΔG_bind_). To have a consensus prediction, several webservers were used, namely SAAMBE-3D^29^, MutaBind2^30^ and mCSM-PPI2^31^. The OXTR/OXTR A218T dimeric models were used as input structure for all webservers and the Thr218 mutation was reverted to WT Ala. It should be noted that the mutant OXTR/OXTR dimer models contain two T218 residues, one in each monomer. The MutaBind2^30^ webserver allows to calculate ΔΔG_bind_ for the double mutant T218A/T218A’. However, both the SAAMBE-3D^29^ and mCSM-PPI2^31^ webservers are limited to a single mutation at a time, either T218A or T218A’. Nonetheless, the change in binding free energy for the double mutant can still be estimated as the sum of the ΔΔG_bind_ of the single mutants^32^, i.e. ΔΔG_bind_ (T218A/T218A’) = ΔΔG_bind_ (T218A) + ΔΔG_bind_ (T218A’). This assumes that the two single mutations (T218A and T218A’) are functionally independent, such that the coupling or interaction free energy between the two residues (218 and 218') is zero. Nonetheless, this additivity approximation might not hold if residues A218 and A218’ (or T218 and T218’) are close at the dimer interface (i.e. C_β_-C_β_ distance < 5.5 Å). Therefore, the values reported here must be taken as a qualitative estimation only.

**Results**

*OXTR monomer structural features.* We generated models of WT and A218T monomers in an active state, one of the intermediate states and an inactive state, using both a single template (with SwissModel^8^) and a multitemplate approach (retrieved from GPCRdb^11^). The models are listed in Tab. S5 and shown in Fig. S2.

Although the models show some differences in the extracellular and intracellular loops (Fig. S2), the seven transmembrane (7TM)-helix bundle (where WT A218 or the T218 variant are located) turns out to be remarkably similar using the two modeling approaches (7TM-C_α_ RMSD *wrt* to the OXTR A218T inactive structure ranging from 2.1 to 3.6 Å). Moreover, the 7TM-C_α_ RMSD between our HM of the inactive state (built with SwissModel using as template the orexin 1 receptor) and the crystal structure of OXTR A218T is 1.78 Å, despite their sequence identity being below 35%. Therefore, the active and intermediate state models, also built using templates with low sequence identity, are expected to be of sufficient quality to predict the structural effects of the A218T variant on the OXTR monomer in different activation states.

Residue 218 is located on the external surface of the fifth transmembrane helix (TM5, see Fig. S2). TM5 is known not to move significantly during receptor activation in class A GPCRs^21, 22^. Consistently, the active, inactive and intermediate state models of the OXTR monomer show a similar position of the TM5 helix (TM5-C_α_ RMSD ranging from 2.1 to 2.9 Å). Hence, the changes in intermolecular interactions between the A218T variant and WT described in the main text (see Fig. 3) are maintained on passing from the inactive to the intermediate and to the active state.


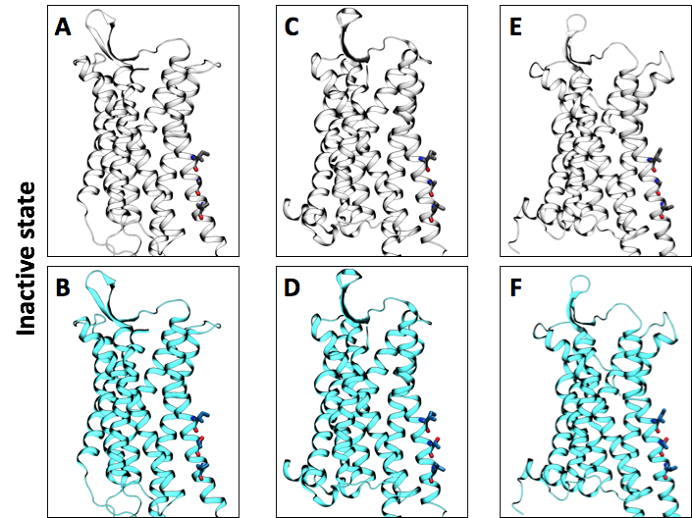


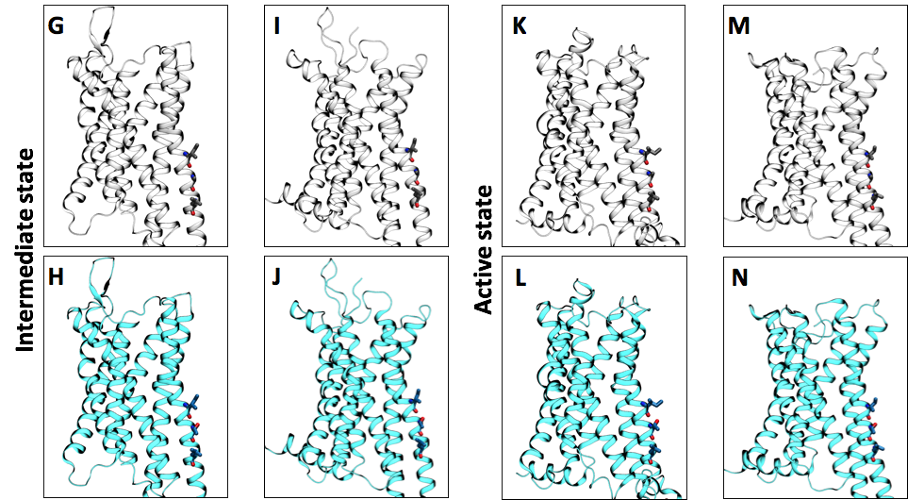


**Fig. S2: WT (gray) and A218T (cyan) OXTR monomeric models in different activation states.** Inactive state models, based either on the crystal structure of Waltenspühl and coworkers^7^ (**A**-**B**), or generated with HM (**C**-**D**), or obtained from GPCRdb^11^ (**E**-**F**). Intermediate state models, based on HM (**G**-**H**) or obtained from GPCRdb (**I**-**J**). Active state models, based on HM (**K**-**L**) or obtained from GPCRdb (**M**-**N**). Residue 218, along with Ile214 (located one helical turn above on TM5) and Leu222 (one helical turn below), are displayed in sticks.

*OXTR monomer stability.* The effect of the A218T variant on monomer stability for all A218T models in Fig. S2 was estimated, at a qualitative level, by calculating the change in protein folding free energy (ΔΔG_fold_, see Tab. S7). As expected^23, 24^, the absolute ΔΔG_fold_ values vary across different webservers. Nonetheless, all of them consistently predict an increase in stability in terms of protein folding free energy of the OXTR A218T mutant compared to WT, regardless of the modeling approach or the receptor activation state used.

*OXTR monomer flexibility.* The effect of the A218T variant on the monomer flexibility for all A218T models in Fig. S2 was estimated, at a qualitative level, by calculating the change in vibrational entropy (ΔΔS_vib_, see Tab. S7). The DynaMut webserver^26^ predicts a decrease in flexibility of the OXTR A218 mutant compared to the WT. This is in line with previous computational studies showing that the presence of Ser/Thr inside TM helices affects their flexibility and dynamics^33^.

**Tab. S7:** **Predicted changes in OXTR monomer stability (ΔΔG_fold_) and flexibility (ΔΔS_vib_) associated to the T218A variant.**

| **OXTR A218T monomer^*^** | **state** | **ΔΔG_fold_**  **(kcal/mol)^#^** | | | | **ΔΔS_vib_ (kcal/mol·K)^§^** |
| --- | --- | --- | --- | --- | --- | --- |
|  |  | **mCSM-**  **membrane** | **DynaMut** | **DynaMut2** | **PremPS** | **DynaMut** |
| model B | inactive | -1.084 | -0.191 | -0.24 | 0.54 | 0.238 |
| model D |  | -0.748 | -0.214 | -0.23 | 0.40 | 0.268 |
| model F |  | -0.887 | -0.172 | -0.20 | 0.20 | 0.215 |
| model H | intermediate | -0.806 | -0.156 | -0.26 | 0.12 | 0.195 |
| model J |  | -1.082 | -0.189 | -0.21 | 0.41 | 0.236 |
| model L | active | -0.771 | -0.165 | -0.16 | 0.29 | 0.206 |
| model M |  | -0.954 | -0.266 | -0.25 | 0.46 | 0.332 |

^*^ The labels refer to the panels shown in Fig. S2; all the OXTR A218T models were used as input of the different webservers and the Thr218 mutation was reverted to WT Ala.

^#^ A negative sign of ΔΔG_fold_ suggests a decrease in stability of OXTR WT compared to the A218T variant, except for PremPS (where a positive sign indicates a destabilizing effect, opposite to the other webservers). For the DynaMut webserver, only the Normal Mode Analysis-based prediction of ΔΔG_fold_ is reported.

^§^ A positive sign of ΔΔS_vib_ predicts an increase in flexibility of OXTR WT relative to the A218T variant.

*OXTR/OXTR dimer structural features.* Based on the experimental GPCR dimer structures in Tab. S6, fourteen OXTR/OXTR TM5/TM5’ homodimer models were built for OXTR A218T, as well as the corresponding fourteen WT OXTR models. Although other dimer interfaces are possible in class A GPCRs^14, 15^ and OXTR^6^, the residue in position 218 is located at TM5, and thus the variant is expected to affect more directly the TM5/TM5’ dimers, in which it is part of the protein/protein interface. All dimer models considered here were based on the OXTR monomer model in the inactive state. TM5 remains mostly immobile during receptor activation^21, 22^ and thus the TM5/TM5’ interface of the OXTR/OXTR models is not expected to change significantly with the activation state of the receptor.

The OXTR dimer models were analyzed with the PRODIGY-CRYSTAL^18, 19^ and PRODIGY^16, 17^ webserver-tools. The results obtained are shown in Tab. S8 and S9 for A218T and WT models, respectively. Unlike the structural templates in Tab. S6, seven models (including the CXCR4-based model, similar to the one in reference^6^) were predicted to be crystallographic-only complexes and thus were not considered for further analysis. The remaining seven models were classified as likely to correspond to biological complexes and exhibit favorable protein/protein binding free energy (Tab. S8 and S9).

**Tab. S8: Mutant (Thr218) OXTR dimer models involving the TM5-TM5’ interface based on structural superposition with GPCR dimer experimental structures in Tab. S6.**

| **Mutant OXTR dimer model** | **Template**  **PDB code** | **Number of interactions^*^** | **Interface**  **Prediction^#^** | **ΔG_bind_**  **(kcal/mol)**^§^ |
| --- | --- | --- | --- | --- |
| OPS-01 | 2Z73 | 24 | XTAL (0.9) | -4.2 |
| OPS-02 | 2Z73 | 33 | XTAL (0.58) | -4.4 |
| CXCR4 | 3ODU | 45 | XTAL (0.828) | -5.7 |
| ADRB1-01 | 4BVN | 44 | XTAL (0.796) | -6.0 |
| OPRM | 4DKL | 186 | BIO (1.0) | -16.5 |
| P2Y12 | 4NTJ | -- | -- | -- |
| ADRB1-02 | 5A8E | 44 | XTAL (0.796) | -6.0 |
| ADRB2 | 5JQH | 72 | BIO (0.612) | -8.7 |
| AA2AR-01 | 5NM4 | 48 | BIO (0.688) | -5.0 |
| AA2AR-02 | 5OLO | 49 | BIO (0.764) | -5.0 |
| AA1R | 5UEN | 41 | BIO (0.616) | -5.2 |
| PTAFR | 5ZKQ | 17 | XTAL (0.568) | -4.4 |
| PE2R3 | 6AK3 | 63 | BIO (0.72) | -7.1 |
| 5HT2C | 6BQG | 95 | BIO (0.972) | -8.6 |

^*^ Number of pair-wise residue contacts, i.e. atom pairs of the two monomers closer than 5.0 Å, obtained with the PRODIGY-CRYSTAL webtool^18, 19^.

^#^ The dimer interface is predicted to be biological (BIO) or crystallographic (XTAL) using the PRODIGY-CRYSTAL webtool^18, 19^. The probability of the classification is indicated between parentheses, with values closer to one indicating the likelihood of the dimer to belong to the BIO or XTAL class, i.e. biological or crystallographic complex, respectively.

^§^ Calculated with the PRODIGY webserver^16, 17^.

**Tab. S9: Wildtype (Ala218) OXTR dimer models involving the TM5-TM5’ interface based on the models in Tab. S8 upon reversal of the mutation Thr218.**

| **Wild-type OXTR dimer model** | **Template**  **PDB code** | **Number of interactions^*^** | **Interface**  **Prediction^#^** | **ΔG_bind_ (kcal/mol)**^§^ |
| --- | --- | --- | --- | --- |
| OPS-01 | 2Z73 | 24 | XTAL (0.9) | -4.5 |
| OPS-02 | 2Z73 | 33 | XTAL (0.58) | -4.2 |
| CXCR4 | 3ODU | 45 | XTAL (0.828) | -5.7 |
| ADRB1-01 | 4BVN | 44 | XTAL (0.796) | -6.0 |
| OPRM | 4DKL | 184 | BIO (0.992) | -15.1 |
| P2Y12 | 4NTJ | -- | -- | -- |
| ADRB1-02 | 5A8E | 44 | XTAL (0.796) | -6.0 |
| ADRB2 | 5JQH | 72 | BIO (0.612) | -8.6 |
| AA2AR-01 | 5NM4 | 48 | BIO (0.688) | -4.9 |
| AA2AR-02 | 5OLO | 49 | BIO (0.764) | -5.0 |
| AA1R | 5UEN | 41 | BIO (0.616) | -5.2 |
| PTAFR | 5ZKQ | 17 | XTAL (0.568) | -4.3 |
| PE2R3 | 6AK3 | 63 | BIO (0.72) | -7.1 |
| 5HT2C | 6BQG | 95 | BIO (0.972) | -8.1 |

^*^ Number of pair-wise residue contacts, i.e. atom pairs of the two monomers closer than 5.0 Å, obtained with the PRODIGY-CRYSTAL webtool^18, 19^.

^#^ The dimer interface is predicted to be biological (BIO) or crystallographic (XTAL) using the PRODIGY-CRYSTAL webtool^18, 19^. The probability of the classification is indicated between parentheses, with values closer to one indicating the likelihood of the dimer to belong to the BIO or XTAL class, i.e. biological or crystallographic complex, respectively.

^§^ Calculated with the PRODIGY webserver^16, 17^.

The top three OXTR/OXTR dimeric models, in terms of ΔΔG_bind_ and number of atom pair contacts across the dimer interface (Tab. S8 and S9), are based on the μ-opioid (OPRM), β2-adrenergic (ADRB2) and serotonin 5-HT_2C_ (5HT2C) receptor templates (see Fig. S3). Interestingly, OPRM belongs to the same peptide receptor family as OXTR^34^, and ADRB2 and 5HT2C have been shown to form heterodimers with OXTR^35-37^. Although all three dimer models exhibit a TM5/TM5’ interface, the relative orientation of the two TM5 helices is different, almost parallel for the ADRB2- and 5HT2C-based models or forming a ~30 degree angle in the OPRM-based model. This different arrangement results in different interhelical interactions in the three dimer models, in particular around residue 218. In the OPRM-based model, residue 218 of one monomer is within 5.5 Å of residues A217 (also located on TM5) and F278 and L282 (TM6) of the adjacent monomer. The A218T mutation is expected to strengthen the interactions with the other monomer, because of the bulkier side chain of Thr compared to Ala. Interestingly, in the 5HT_2C_-based model WT Ala218 does not have any neighbor residue from the adjacent monomer, but mutation to Thr218 allows to establish an additional interaction with the adjacent monomer, i.e. with L255 on TM4. Instead, residue 218 does not seem to form any interhelical interaction in the ADRB2-based models, either WT or A218T. In summary, two out of our top three dimer models predict an increase in dimer interactions of the A218T mutant compared to WT OXTR.


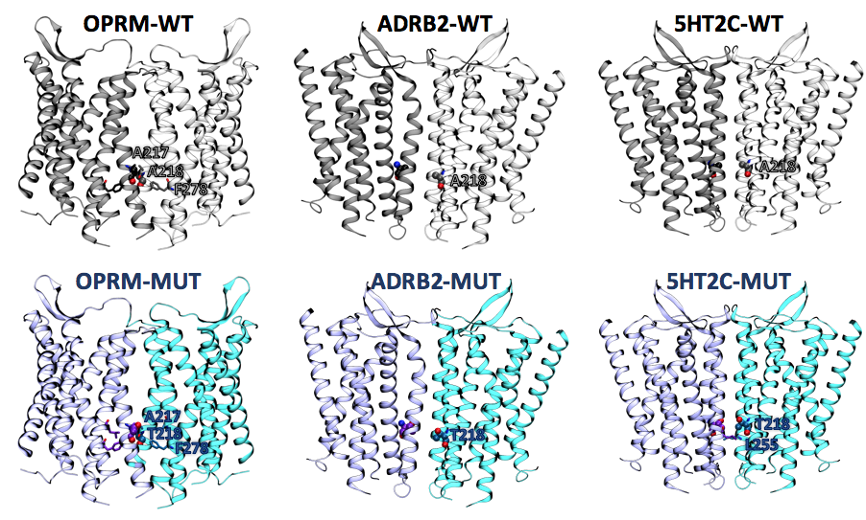


**Fig. S3: Comparison of top three OXTR/OXTR dimer models with a TM5/TM5’ interface, based on the experimental structures of the mu-opioid (OPRM), the beta2-adrenergic (ADRB2) and the serotonin 2C receptor (5HT2C) dimers.** Residue 218 is shown as spheres and residues within 5.5 Å of A218/T218 as sticks.

*OXTR/OXTR dimer stability.* Tab. S10 shows the predicted change in protein-protein binding energy upon mutation for the seven OXTR/OXTR dimer models classified as biological (see Tab. S8). Although the absolute values vary across different webservers, they consistently predict an increase in dimer stability of the A218T variant compared to WT.

**Tab. S10:** **Change in protein-protein binding free energy upon mutation (ΔΔG_bind_).** Only the seven OXTR A218T dimer models that were predicted to be biological complexes (see Tab. S8) were analyzed.

| **OXTR dimer model** | **Template**  **PDB code** | **A218 at interface?^*^** | **ΔΔG_bind_^SAAMBE-3D^ (kcal/mol)^#,§^** | **ΔΔG_bind_^MutaBind2^ (kcal/mol)**^#^**^,§^** | **ΔΔG_bind_^mCSM-PPI2^ (kcal/mol)^#,§^** |
| --- | --- | --- | --- | --- | --- |
| OPRM | 4DKL | yes (4.56) | 1.52 | 0.17 | -0.804 |
| ADRB2 | 5JQH | no (7.63) | 0.38 | 0.21 | -0.220 |
| AA2AR-01 | 5NM4 | no (8.77) | 0.38 | 0.21 | -0.189 |
| AA2AR-02 | 5OLO | no (8.11) | 0.38 | 0.23 | -0.193 |
| AA1R | 5UEN | no (9.06) | 0.38 | 0.27 | -0.269 |
| PE2R3 | 6AK3 | no (8.37) | 0.38 | 0.18 | -0.262 |
| 5HT2C | 6BQG | yes (5.36) | 0.38 | 0.22 | -0.142 |

^*^ Residue 218 is considered to be at the interface if it is within 5.5 Å of the adjacent monomer and shows lower residue solvent accessibility in the OXTR-OXTR dimer than in the individual monomer, according to the MutaBind2 webserver. The distance to the interface (in Å) is indicated in parentheses.

^#^ A positive value of ΔΔG_bind_ indicates a destabilizing effect of WT A218 relative to the T218 variant, except for the mCSM-PPI2 web server (where a negative sign indicates a destabilizing effect, opposite to the other webservers).

^§^ The ΔΔG_bind_ values reported for SAAMBE-3D and mCSM-PPI2 correspond to the sum of the T218A and T218A’ changes (see SI Methods). Instead, the MutaBind2 webserver calculates directly ΔΔG_bind_ for the double mutant T218A/T218A’.

Taken together, these results suggest a larger stability of A218T relative to WT, in both monomers and dimers. Both forms are expected to co-exist at the expression conditions of the experiments. Thus, our finding may be in line with the cycloheximide assays reported in the main text (Fig. 1d). Nonetheless, this point should be taken with caution, as our calculations provide a (qualitative) estimation of thermodynamics stabilities, which differs from the experimental readout, related to protein degradation. Moreover, the calculations presented above account only in a very simplified way for the contribution of protein dynamics, by using several OXTR structural models, together with normal mode-based sampling in the case of DynaMut^26^. Extensive molecular dynamics (MD) simulations of the OXTR in different conformational states would be needed in order to properly sample the mutational effects on protein flexibility. Furthermore, the webservers used here employ simplified energy- and machine learning-based functions to (qualitatively) estimate the mutational effects on protein stability. A more accurate estimation of such effects would require the use of MD simulations in combination with either energy decomposition approaches^38-40^ or free energy perturbation calculations^41^.

## Mathematical modeling of the OXTR-mediated signaling pathway.

*OXT-triggered signaling pathways.* OXTRs are able to couple to different G-proteins. OXTR-mediated activation of G_αq_ , G_αo_ , and G_αi_ -proteins affect, together with the G_βγ_ subunit, a diversity of signaling cascades (such as the PLC, PKA, PKC, DAG kinase, MAPK, CaMK, and PI4-Kinase-Rho pathways), as well as membrane ion channels (conducting calcium, potassium and sodium)^42, 43^. These pathways will, consequently, converge on the activation of transcription factors like CREB or MEF-2, which, depending on the G-protein coupling, can transduce growth-inhibitory or proliferatory signals^42^.

The specific coupling of the different G proteins to the receptor, and consequently the triggered physiological effect, is determined by the expression level of the individual G proteins and the local ligand concentration^44^. Busnelli *et al.* have demonstrated that the G_q_-mediated pathways are the first to be triggered at low OXT concentrations, whereas activation of the G_i/o_-mediated pathways needs at least a 10-fold higher OXT concentration^45^. Indeed, it has been proposed that, at such high OXT concentrations, all the activated G-proteins may act synergistically, causing a rapid desensitization of the OXTR^46^.

At low concentrations values of OXT^45^ the G_q_-mediated pathway is triggered. Both the G_q_-α and G_q_-βγ subunits activate phospholipase C beta (PLC-β), which consequently generates inositol-1,4,5-trisphosphate (IP_3_) and diacylglycerol (DAG) from phosphatidylinositol 4,5-bisphosphate (PIP_2_). IP_3_, as well as other phospholipid metabolites, are implicated in the release of Ca^2+^ from internal stores, especially from the endoplasmic reticulum (ER). This has been measured here using Ca^2+^ imaging for both WT and A218T cells in a Ca^2+^-free Ringer medium (Fig. 2). The IP_3_-dependent release of Ca^2+^ from the ER is mediated by IP_3_ receptors (IP_3_Rs) expressed on the organelle membrane. However, this Ca^2+^ release mechanism is also governed by the Ca^2+^ itself, making this dual activation of IP_3_Rs central for the oscillatory nature of the intracellular concentration of Ca^2+47^. DAG, together with Ca^2+^, activates the phosphokinase C (PKC) that, in turn, activates the MAPK pathway^42^, in particular phosphorylation of ERK1/2, which has been measured here by western blot (Fig. 1).

At higher OXT concentrations^45^, the G_i/o_ proteins coupled to the OXTR are also activated, affecting kinase pathways and membrane ion channels’ activity. Some G_i/o_-α subunit isoforms and G_i/o_-βγ subunits directly inhibit adenylyl cyclase, reducing the intracellular levels of cAMP and affecting the activation of PKA. In addition, the G_i/o_-α subunits can also interfere with PKA function indirectly, by altering its nuclear translocation^43^. Although not yet demonstrated in mammalian cells, evidence in *C. elegans* indicated that the G_o_-α subunits trigger a decrease in the levels of DAG, acting on the DAG kinase pathway. Since activation of PKC is highly dependent on DAG, G_o_-α subunits thus indirectly inhibit PKC^43^. Taking also into account that phosphorylation of IP_3_Rs by PKA and PKC plays an important role in modulation of IP_3_-induced Ca^2+^ signals^48^, the effect on PKA/C by activation of G_i/o_ proteins might not affect the generation and regulation of IP_3_-induced Ca^2+^ signals by IP_3_Rs, but rather have a modulatory effect^43, 49^.

The direct activation of PLC-β by G_i/o_ proteins has been shown to be mediated through the βγ subunit. However, the general rapid desensitization of the G_i/o_ signaling implies that the concentration of IP_3_ generated by PLC-β activation mediated by G_i/o_-βγ subunit will be too low to generate a robust Ca^2+^-release from ER^46^.

Altogether, activation of G_i/o_ proteins may have a very limited contribution to the IP_3_-mediated Ca^2+^ signals at the ER. Moreover, the very low expression of G_o_ proteins in HEK293 cells^50^, used in the experiments presented here, may further minimize their contribution. Therefore, in our experimental setup, regulation of the IP_3_-mediated Ca^2+^ signals at the ER is probably mainly due to the activation of the G_q_-mediated pathway.

*Systems biology modeling of the Gq-activated Ca^2+^ signals at the ER.* The cascade from OXT-triggered G_q_ activation to the downstream Ca^2+^ release from the ER through IP_3_R was modeled by integrating two previously existing mathematical models: (i) a model of the activation of the eukaryotic MAPK pathway (via PKC/Raf-1) upon serotonin 2A receptor activation^51^; and (ii) a dynamic model of the IP_3_R action, with a change in Ca^2+^ and IP_3_ concentrations resulting in intracellular Ca^2+^ oscillations^52^. The equations and kinetic constants for each of the reactions of the systems biology model developed here are shown in Tab. S12. Namely, we selected from model (i)^51^ the reactions upstream from the production of IP_3_ by PLC-β. This is the crossroad with model (ii)^52^, which dynamically simulates the IP_3_ and Ca^2+^-dependent Ca^2+^ release from the ER into the cytosol. In particular, Keizer and De Young proposed a simple model^52^ to account for both IP_3_ stimulation and Ca^2+^ inhibition by considering the IP_3_R as a homotetramer, i.e. one binding site for IP_3_ and one for the Ca^2+^. Each monomer can thus exist in one of four states: S_0_ (unbound), S_1_ (bound to IP_3_), S_2_ (bound to Ca^2+^), and S_3_ (bound to IP_3_ and Ca^2+^); however, channel opening requires all four subunits to be in state S_1_. Under such assumptions, Keiser and De Young^52^ defined the flux of Ca^2+^ through the IP_3_R as proportional to the open probability for one IP_3_R at equilibrium. Considering that the species in each deterministic reaction of our model are expressed in concentration values (Tab. S13), we defined the IP_3_R open probability as the equilibrium fraction of subunits in state S_1_ (see [IP3R_IP3] in reaction 22 in Tab. S12).

*Modeling of the Ca^2+^ concentration differences between WT and A218T OXTR.* The systems biology model described above was used to rationalize the experimentally measured Ca^2+^ concentrations with 100 nM OXT and in the absence of extracellular Ca^2+^ (Fig. 2e, blue bars). Simulation parameters and initial concentrations of the model are provided in Tab. S11 and S13. Since the molecular modeling results suggested that the A218T variant affects receptor activation, we modified the forward kinetic constant of reaction 3 in Tab. S12. This parameter describes G_q_-protein binding to the receptor, which implicitly depends on receptor activation. We introduced a scaling factor (Tab. S11), so that this kinetic constant remains unmodified for WT OXTR (*kf_coupling_wt* = 1), whereas it is adjusted for the A218T mutant (*kf_coupling_mut* = variable) to attempt to simulate the experimentally observed higher Ca^2+^ signal in A218T compared with WT cells incubated in Ca^2+^-free Ringer (Fig. 2e).

By simply scaling the *kf_coupling_mut* by a factor of 1.45, our systems biology scheme is able to reproduce the shape of the experimental Ca^2+^ concentration curves (Fig. 2d) and the ratio between the WT and A218T OXTR amplitudes. The calculated WT/A218T ratios for the AUC and full width at half maximum (Fig. 2h and 2j) are also in agreement with the experimental results (Fig. 2g and 2i).

*Limitations and perspectives of the systems biology model*. Although our model is able to qualitatively predict a relative change in the concentration of Ca^2+^ comparable with the experimental data, one should keep in mind that it was developed based on two previous independent models. Some of the kinetic constants in those models were tuned in order to mimic specific experimental data for other GPCRs. Hence, future improvements of our model should involve fitting of kinetic constants to experimental data obtained for the OXTR signaling pathway. Moreover, since the activation of the MAPK/ERK pathway depends also on the extracellular Ca^2+^ and on the activation of the epidermal growth factor receptor (EGFR), further extensions of our model should incorporate these secondary pathways. Finally, our systems biology model assumes a monomeric OXTR as the starting point of the signaling pathway. However, OXTR exists in homo- and hetero-dimeric forms, as discussed above. Therefore, future improvements of the cascade description need to include this possibility.

**Tab. S11:** **Simulation Parameters.**

| **Parameter** | **Value** | **Description** |
| --- | --- | --- |
| ttotal | 250 s | total simulation time |
| nsteps | 10000 | number integration steps |
| time_in, time_out | 50 s , 51s | time of the simulation in which OXT can bind to OXTR |
| kf_coupling_wt, kf_coupling_mut | 1, variable | Scaling factor that multiplies the forward kinetic constant of reaction 3 in Tab. S12.  See text for more details. |

**Tab. S12:** **Equations and kinetic constants for each of the reactions of the systems biology model developed here.**


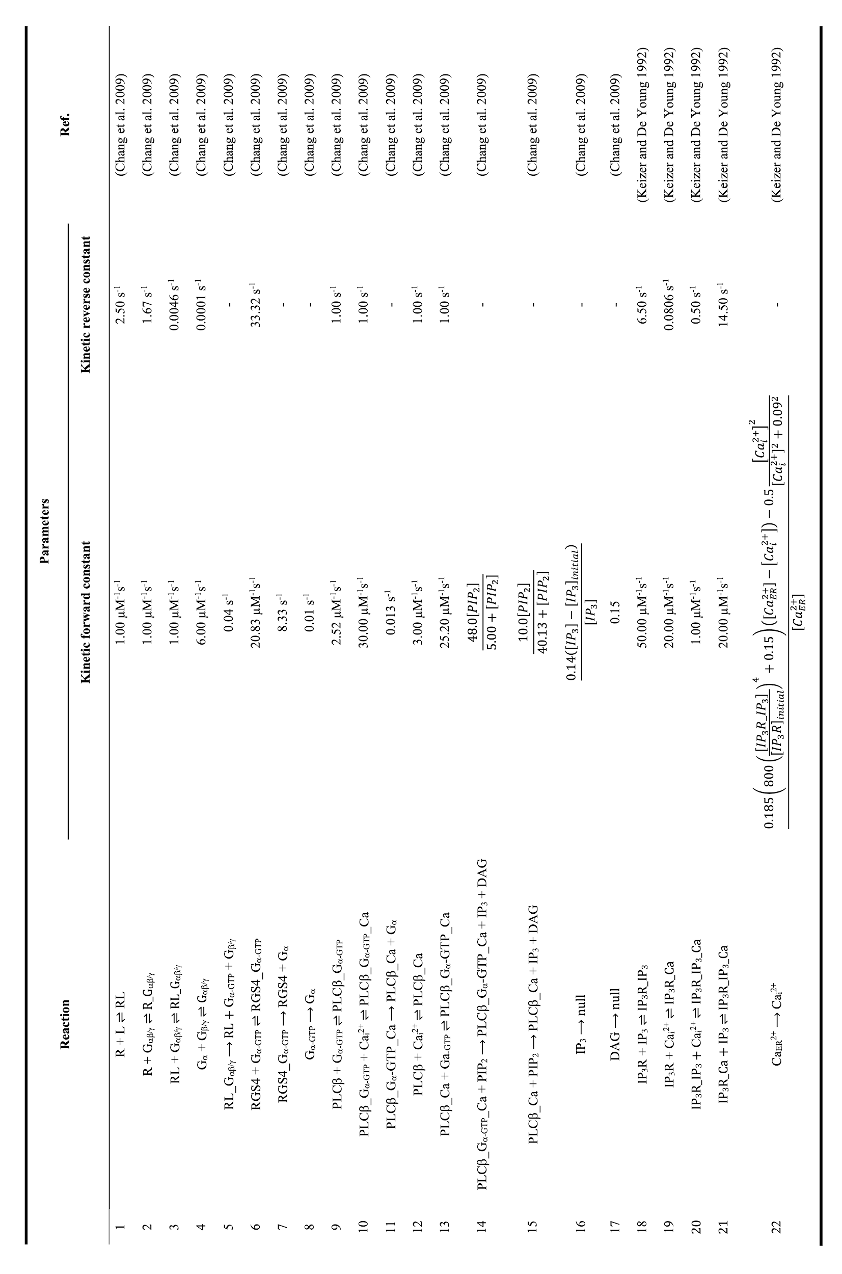


**Tab. S13:** **Initial concentrations of each of the species in the mathematical model.**

| **Parameter** | **Value** **(µM)** | **Reference** |
| --- | --- | --- |
| R_init_ | 1.4107 | (Chang et al. 2009) |
| L_init_ | 0.1 | OXT concentration used in the experiments presented in this work |
| G_init_ | 0.0027739 | (Chang et al. 2009) |
| Gα-GTP_init_ | 6.4172E-4 | (Chang et al. 2009) |
| Gβγ_init_ | 0.0037173 | (Chang et al. 2009) |
| R_Gαβγ_init_ | 0.27*Rinit | (Chang et al. 2009) |
| Gαβγ_init_ | 0.61869 | (Chang et al. 2009) |
| RGS4_init_ | 0.019994 | (Chang et al. 2009) |
| RGS4_Gα-GTP_init_ | 6.4168E-6 | (Chang et al. 2009) |
| Ca_i_^2+^_init_ | 0.1^*^ | (Keizer and De Young 1992) |
| Ca_ER_^2+^_init_ | 1 | - |
| PLCβ_init_ | 0.090022 | (Chang et al. 2009) |
| PLCβ_Gα_GTP_init_ | 1.4492E-4 | (Chang et al. 2009) |
| PLCβ_Ca_init_ | 0.0093825 | (Chang et al. 2009) |
| PLCβ_Ca_G_GTP_init_ | 1.5038E-4 | (Chang et al. 2009) |
| PIP_2init_ | 2.6578 | (Chang et al. 2009) |
| IP_3init_ | 0.21952 | (Chang et al. 2009) |
| DAG_init_ | 0.055555 | (Chang et al. 2009) |
| IP_3_R_init_ | 0.119 | (Taylor and Konieczny 2016) |

*Value chosen according to the resting value of cytosolic calcium concentration present in typical experiments for a variety of cells (50-100 nM)^52^ that ensures the correct parameters for the reaction 22 of Tab. S12.

**References Supplementary Information**

1. Grote S. GOfuncR: Gene ontology enrichment using FUNC. R package version 1.8.0. 2020.

2. van den Burg EH, Stindl J, Grund T, Neumann ID, Strauss O. Oxytocin Stimulates Extracellular Ca2+ Influx Through TRPV2 Channels in Hypothalamic Neurons to Exert Its Anxiolytic Effects. *Neuropsychopharmacology* 2015; **40**(13)**:** 2938-2947.

3. Meyer M, Berger I, Winter J, Jurek B. Oxytocin alters the morphology of hypothalamic neurons via the transcription factor myocyte enhancer factor 2A (MEF-2A). *Mol Cell Endocrinol* 2018; **477:** 156-162.

4. Meyer M, Kuffner K, Winter J, Neumann ID, Wetzel CH, Jurek B. Myocyte Enhancer Factor 2A (MEF2A) Defines Oxytocin-Induced Morphological Effects and Regulates Mitochondrial Function in Neurons. *Int J Mol Sci* 2020; **21**(6).

5. Cottet M, Albizu L, Perkovska S, Jean-Alphonse F, Rahmeh R, Orcel H *et al.* Past, present and future of vasopressin and oxytocin receptor oligomers, prototypical GPCR models to study dimerization processes. *Curr Opin Pharmacol* 2010; **10**(1)**:** 59-66.

6. Busnelli M, Kleinau G, Muttenthaler M, Stoev S, Manning M, Bibic L *et al.* Design and Characterization of Superpotent Bivalent Ligands Targeting Oxytocin Receptor Dimers via a Channel-Like Structure. *J Med Chem* 2016; **59**(15)**:** 7152-7166.

7. Waltenspuhl Y, Schoppe J, Ehrenmann J, Kummer L, Pluckthun A. Crystal structure of the human oxytocin receptor. *Sci Adv* 2020; **6**(29)**:** eabb5419.

8. Waterhouse A, Bertoni M, Bienert S, Studer G, Tauriello G, Gumienny R *et al.* SWISS-MODEL: homology modelling of protein structures and complexes. *Nucleic Acids Res* 2018; **46**(W1)**:** W296-W303.

9. Dunbrack RL, Jr. Rotamer libraries in the 21st century. *Curr Opin Struct Biol* 2002; **12**(4)**:** 431-440.

10. Pettersen EF, Goddard TD, Huang CC, Couch GS, Greenblatt DM, Meng EC *et al.* UCSF Chimera--a visualization system for exploratory research and analysis. *J Comput Chem* 2004; **25**(13)**:** 1605-1612.

11. Pandy-Szekeres G, Munk C, Tsonkov TM, Mordalski S, Harpsoe K, Hauser AS *et al.* GPCRdb in 2018: adding GPCR structure models and ligands. *Nucleic Acids Res* 2018; **46**(D1)**:** D440-D446.

12. Remmert M, Biegert A, Hauser A, Soding J. HHblits: lightning-fast iterative protein sequence searching by HMM-HMM alignment. *Nat Methods* 2011; **9**(2)**:** 173-175.

13. Lemel L, Niescierowicz K, Garcia-Fernandez MD, Darre L, Durroux T, Busnelli M *et al.* The ligand-bound state of a G protein-coupled receptor stabilizes the interaction of functional cholesterol molecules. *J Lipid Res* 2021**:** 100059.

14. Garcia-Recio A, Navarro G, Franco R, Olivella M, Guixa-Gonzalez R, Cordomi A. DIMERBOW: exploring possible GPCR dimer interfaces. *Bioinformatics* 2020; **36**(10)**:** 3271-3272.

15. Stenkamp RE. Identifying G protein-coupled receptor dimers from crystal packings. *Acta Crystallogr D Struct Biol* 2018; **74**(Pt 7)**:** 655-670.

16. Vangone A, Bonvin AM. Contacts-based prediction of binding affinity in protein-protein complexes. *Elife* 2015; **4:** e07454.

17. Xue LC, Rodrigues JP, Kastritis PL, Bonvin AM, Vangone A. PRODIGY: a web server for predicting the binding affinity of protein-protein complexes. *Bioinformatics* 2016; **32**(23)**:** 3676-3678.

18. Elez K, Bonvin A, Vangone A. Distinguishing crystallographic from biological interfaces in protein complexes: role of intermolecular contacts and energetics for classification. *BMC Bioinformatics* 2018; **19**(Suppl 15)**:** 438.

19. Jimenez-Garcia B, Elez K, Koukos PI, Bonvin AM, Vangone A. PRODIGY-crystal: a web-tool for classification of biological interfaces in protein complexes. *Bioinformatics* 2019; **35**(22)**:** 4821-4823.

20. Meng EC, Pettersen EF, Couch GS, Huang CC, Ferrin TE. Tools for integrated sequence-structure analysis with UCSF Chimera. *BMC Bioinformatics* 2006; **7:** 339.

21. Roth CB, Hanson MA, Stevens RC. Stabilization of the human beta2-adrenergic receptor TM4-TM3-TM5 helix interface by mutagenesis of Glu122(3.41), a critical residue in GPCR structure. *J Mol Biol* 2008; **376**(5)**:** 1305-1319.

22. Zhou Q, Yang D, Wu M, Guo Y, Guo W, Zhong L *et al.* Common activation mechanism of class A GPCRs. *Elife* 2019; **8**: e50279.

23. Strokach A, Corbi-Verge C, Kim PM. Predicting changes in protein stability caused by mutation using sequence-and structure-based methods in a CAGI5 blind challenge. *Hum Mutat* 2019; **40**(9)**:** 1414-1423.

24. Pandurangan AP, Blundell TL. Prediction of impacts of mutations on protein structure and interactions: SDM, a statistical approach, and mCSM, using machine learning. *Protein Sci* 2020; **29**(1)**:** 247-257.

25. Pires DEV, Rodrigues CHM, Ascher DB. mCSM-membrane: predicting the effects of mutations on transmembrane proteins. *Nucleic Acids Res* 2020; **48**(W1)**:** W147-W153.

26. Rodrigues CH, Pires DE, Ascher DB. DynaMut: predicting the impact of mutations on protein conformation, flexibility and stability. *Nucleic Acids Res* 2018; **46**(W1)**:** W350-W355.

27. Rodrigues CHM, Pires DEV, Ascher DB. DynaMut2: Assessing changes in stability and flexibility upon single and multiple point missense mutations. *Protein Sci* 2021; **30**(1):60-9.

28. Chen Y, Lu H, Zhang N, Zhu Z, Wang S, Li M. PremPS: Predicting the impact of missense mutations on protein stability. *PLoS Comput Biol* 2020; **16**(12)**:** e1008543.

29. Pahari S, Li G, Murthy AK, Liang S, Fragoza R, Yu H *et al.* SAAMBE-3D: Predicting Effect of Mutations on Protein-Protein Interactions. *Int J Mol Sci* 2020; **21**(7): 2563.

30. Zhang N, Chen Y, Lu H, Zhao F, Alvarez RV, Goncearenco A *et al.* MutaBind2: Predicting the Impacts of Single and Multiple Mutations on Protein-Protein Interactions. *iScience* 2020; **23**(3)**:** 100939.

31. Rodrigues CHM, Myung Y, Pires DEV, Ascher DB. mCSM-PPI2: predicting the effects of mutations on protein-protein interactions. *Nucleic Acids Res* 2019; **47**(W1)**:** W338-W344.

32. Serrano L, Horovitz A, Avron B, Bycroft M, Fersht AR. Estimating the contribution of engineered surface electrostatic interactions to protein stability by using double-mutant cycles. *Biochemistry* 1990; **29**(40)**:** 9343-9352.

33. Del Val C, White SH, Bondar AN. Ser/Thr motifs in transmembrane proteins: conservation patterns and effects on local protein structure and dynamics. *J Membr Biol* 2012; **245**(11)**:** 717-730.

34. Munk C, Isberg V, Mordalski S, Harpsoe K, Rataj K, Hauser AS *et al.* GPCRdb: the G protein-coupled receptor database - an introduction. *Br J Pharmacol* 2016; **173**(14)**:** 2195-2207.

35. Wrzal PK, Devost D, Petrin D, Goupil E, Iorio-Morin C, Laporte SA *et al.* Allosteric interactions between the oxytocin receptor and the beta2-adrenergic receptor in the modulation of ERK1/2 activation are mediated by heterodimerization. *Cell Signal* 2012; **24**(1)**:** 342-350.

36. Wrzal PK, Goupil E, Laporte SA, Hebert TE, Zingg HH. Functional interactions between the oxytocin receptor and the beta2-adrenergic receptor: implications for ERK1/2 activation in human myometrial cells. *Cell Signal* 2012; **24**(1)**:** 333-341.

37. Chruscicka B, Wallace Fitzsimons SE, Borroto-Escuela DO, Druelle C, Stamou P, Nally K *et al.* Attenuation of Oxytocin and Serotonin 2A Receptor Signaling through Novel Heteroreceptor Formation. *ACS Chem Neurosci* 2019; **10**(7)**:** 3225-3240.

38. Morra G, Colombo G. Relationship between energy distribution and fold stability: Insights from molecular dynamics simulations of native and mutant proteins. *Proteins* 2008; **72**(2)**:** 660-672.

39. Montefiori M, Pilotto S, Marabelli C, Moroni E, Ferraro M, Serapian SA *et al.* Impact of Mutations on NPAC Structural Dynamics: Mechanistic Insights from MD Simulations. *J Chem Inf Model* 2019; **59**(9)**:** 3927-3937.

40. Meli M, Morra G, Colombo G. Simple Model of Protein Energetics To Identify Ab Initio Folding Transitions from All-Atom MD Simulations of Proteins. *J Chem Theory Comput* 2020; **16**(9)**:** 5960-5971.

41. Gapsys V, Michielssens S, Seeliger D, de Groot BL. Accurate and Rigorous Prediction of the Changes in Protein Free Energies in a Large-Scale Mutation Scan. *Angew Chem Int Ed Engl* 2016; **55**(26)**:** 7364-7368.

42. Jurek B, Neumann ID. The Oxytocin Receptor: From Intracellular Signaling to Behavior. *Physiol Rev* 2018; **98**(3)**:** 1805-1908.

43. Jiang M, Bajpayee NS. Molecular mechanisms of G_o_ signaling. *Neurosignals* 2009; **17**(1)**:** 23-41.

44. Busnelli M, Chini B. Molecular Basis of Oxytocin Receptor Signalling in the Brain: What We Know and What We Need to Know. *Curr Top Behav Neurosci* 2018; **35:** 3-29.

45. Busnelli M, Sauliere A, Manning M, Bouvier M, Gales C, Chini B. Functional selective oxytocin-derived agonists discriminate between individual G protein family subtypes. *J Biol Chem* 2012; **287**(6)**:** 3617-3629.

46. Van H, II, Oron Y. Go G-proteins mediate rapid heterologous desensitization of G-protein coupled receptors in Xenopus oocytes. *J Cell Physiol* 2005; **204**(2)**:** 455-462.

47. Taylor CW, Konieczny V. IP3 receptors: Take four IP3 to open. *Sci Signal* 2016; **9**(422)**:** pe1.

48. Vermassen E, Fissore RA, Nadif Kasri N, Vanderheyden V, Callewaert G, Missiaen L *et al.* Regulation of the phosphorylation of the inositol 1,4,5-trisphosphate receptor by protein kinase C. *Biochem Biophys Res Commun* 2004; **319**(3)**:** 888-893.

49. Xie R, Li L, Goshima Y, Strittmatter SM. An activated mutant of the alpha subunit of G(o) increases neurite outgrowth via protein kinase C. *Brain Res Dev Brain Res* 1995; **87**(1)**:** 77-86.

50. Atwood BK, Lopez J, Wager-Miller J, Mackie K, Straiker A. Expression of G protein-coupled receptors and related proteins in HEK293, AtT20, BV2, and N18 cell lines as revealed by microarray analysis. *BMC Genomics* 2011; **12:** 14.

51. Chang CW, Poteet E, Schetz JA, Gumus ZH, Weinstein H. Towards a quantitative representation of the cell signaling mechanisms of hallucinogens: measurement and mathematical modeling of 5-HT1A and 5-HT2A receptor-mediated ERK1/2 activation. *Neuropharmacology* 2009; **56 Suppl 1:** 213-225.

52. Keizer J, De Young GW. Two roles of Ca2+ in agonist stimulated Ca2+ oscillations. *Biophys J* 1992; **61**(3)**:** 649-660.

**Figure legends**

**Fig. S1: Uncropped Western Blot images shown in Fig. 1. Blots on the left side show the respective antibody image and blots on the right side the corresponding stain-free blot image for total protein loading control. a,** Uncropped images of total OXTR expression Blot (Fig. 1c). **b,** Uncropped images for blots of the Cycloheximide Assay showing the 3x-FLAG expression (Fig. 1f). **c,** Uncropped images of pERK1/2 blots (Fig. 1g and 1h). **d,** Uncropped images of ERK1/2 total blot. The groups did not show significant differences in the expression of ERK1/2 total.

**Fig. S2: WT (gray) and A218T (cyan) OXTR monomeric models in different activation states.** Inactive state models, based either on the crystal structure of Waltenspühl and coworkers^7^ (**A**-**B**), or generated with HM (**C**-**D**), or obtained from GPCRdb^11^ (**E**-**F**). Intermediate state models, based on HM (**G**-**H**) or obtained from GPCRdb (**I**-**J**). Active state models, based on HM (**K**-**L**) or obtained from GPCRdb (**M**-**N**). Residue 218, along with Ile214 (located one helical turn above on TM5) and Leu222 (one helical turn below), are displayed in sticks.

**Fig. S3: Comparison of top three OXTR/OXTR dimer models with a TM5/TM5’ interface, based on the experimental structures of the mu-opioid (OPRM), the beta2-adrenergic (ADRB2) and the serotonin 2C receptor (5HT2C) dimers.** Residue 218 is shown as spheres and residues within 5.5 Å of A218/T218 as sticks.

**Table legends**

**Tab. S1: Identified insertion points of the oxytocin receptor (OXTR) gene constructs in human HEK293 cells.**

**Tab. S2: Differentially expressed genes close to the insertion points and their p-value.**

**Tab. S3:** **Number (#) of analyzed genes and enriched gene ontology (GO) terms.** GO terms were identified for genes significantly up- and down-regulated in OXTR A218T compared to WT cells separately, as well as in the combined dataset.

**Tab. S4: Selection of gene ontology (GO) terms up- and downregulated in oxytocin receptor A218T *versus* wildtype cells with identification number.**

**Tab. S5: Templates used to build the OXTR monomeric models.**

**Tab. S6: Class A GPCR dimer X-ray structures involving TM5 at the protein/protein interface present in the DIMERBOW^14^ database (version: March 5^th^, 2020).**

**Tab. S7:** **Predicted changes in OXTR monomer stability (ΔΔG_fold_) and flexibility (ΔΔS_vib_) associated to the T218A variant.**

**Tab. S8: Mutant (Thr218) OXTR dimer models involving the TM5-TM5’ interface based on structural superposition with GPCR dimer experimental structures in Tab. S6.**

**Tab. S9: Wildtype (Ala218) OXTR dimer models involving the TM5-TM5’ interface based on the models in Tab. S8 upon reversal of the mutation Thr218.**

**Tab. S10:** **Change in protein-protein binding free energy upon mutation (ΔΔG_bind_).** Only the seven OXTR A218T dimer models that were predicted to be biological complexes (see Tab. S8) were analyzed.

**Tab. S11:** **Simulation Parameters.**

**Tab. S12:** **Equations and kinetic constants for each of the reactions of the systems biology model developed here.**

**Tab. S13:** **Initial concentrations of each of the species in the mathematical model.**

**Chart S1. Chemical structure of retosiban (Vaccinationist, Public domain, via Wikimedia Commons).**
