# Supplementary figures and images for: Structure-function relationships of the disease-linked A218T oxytocin receptor variant
Source: Mol Psychiatry. 2022 Jan 4;27(2):907–17. doi: 10.1038/s41380-021-01241-8 (PMC9054668; doi:10.1038/s41380-021-01241-8)

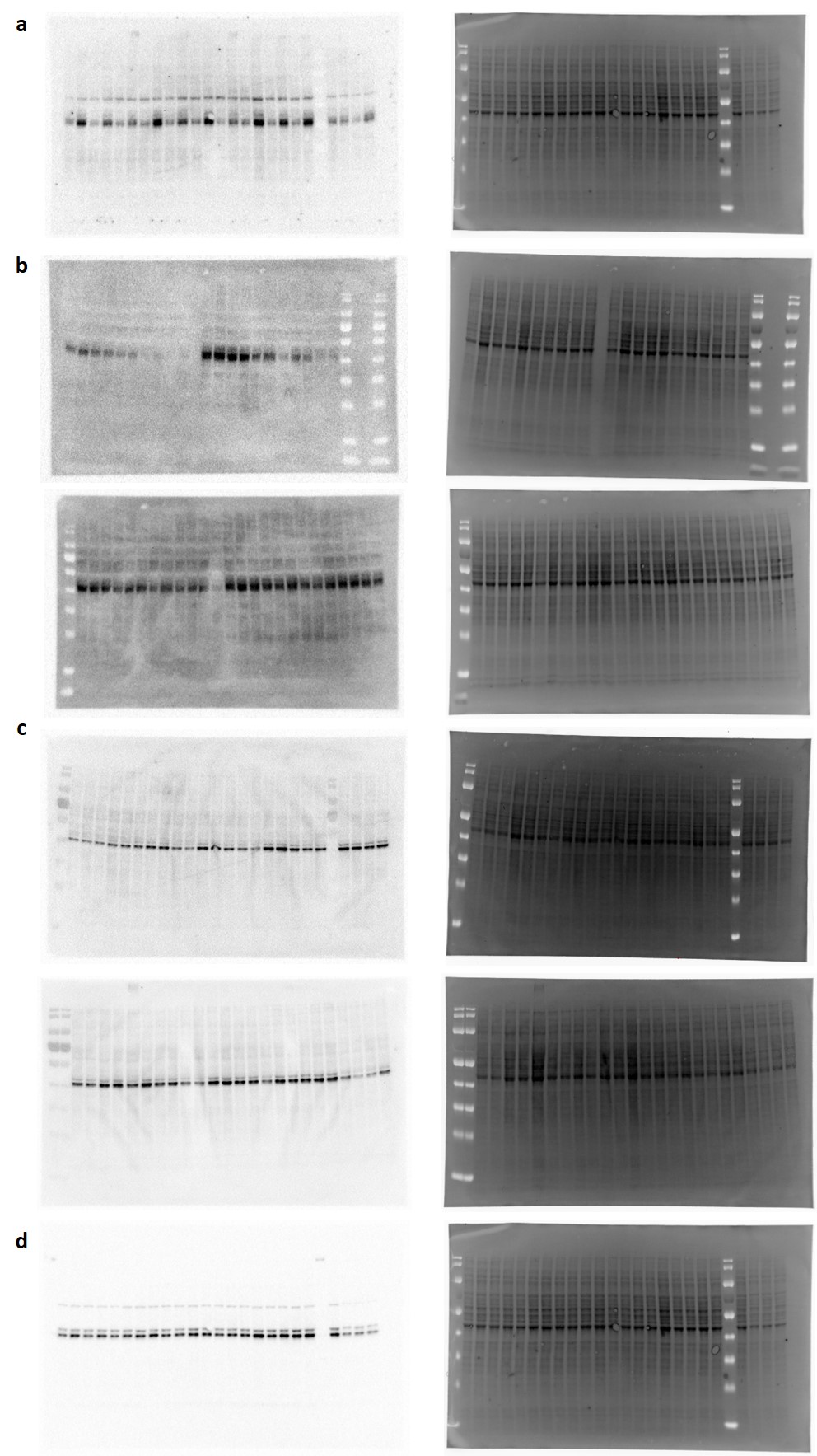

Supplement: Supplementary file 2 — Supplementary Figure S1 [file 41380_2021_1241_MOESM2_ESM.jpg]
